# Decoding dynamic interactions between EGFR‐TKD and DAC through computational and experimental approaches: A novel breakthrough in lung melanoma treatment

**DOI:** 10.1111/jcmm.18263

**Published:** 2024-04-29

**Authors:** Rajesh Kumar Meher, Showkat Ahmad Mir, Kritika Singh, Nobendu Mukerjee, Binata Nayak, Ajoy Kumer, Torki A. Zughaibi, Mohd Shahnawaz Khan, Shams Tabrez

**Affiliations:** ^1^ School of Life Sciences Sambalpur University Burla Odisha India; ^2^ Offenburg University of Applied Sciences Offenburg Germany; ^3^ Center for Global Health Research, Saveetha Medical College and Hospital, Saveetha Institute of Medical and Technical Sciences Chennai India; ^4^ Department of Health Sciences Novel Global Community Educational Foundation Hebersham New South Wales Australia; ^5^ Department of Chemistry College of Arts and Sciences, IUBAT‐International University of Business Agriculture and Technology Dhaka Bangladesh; ^6^ King Fahd Medical Research Center King Abdulaziz University Jeddah Saudi Arabia; ^7^ Department of Medical Laboratory Sciences, Faculty of Applied Medical Sciences King Abdulaziz University Jeddah Saudi Arabia; ^8^ Department of Biochemistry College of Science, King Saud University Riyadh Saudi Arabia

**Keywords:** cancer, CTX‐1, EGFR‐TKD, erlotinib, lung cancer, ROS

## Abstract

In the quest for effective lung cancer treatments, the potential of 3,6‐diaminoacridine‐9‐carbonitrile (DAC) has emerged as a game changer. While DAC's efficacy against glioblastoma is well documented, its role in combating lung cancer has remained largely untapped. This study focuses on CTX‐1, exploring its interaction with the pivotal EGFR‐TKD protein, a crucial target in lung cancer therapeutics. A meticulous molecular docking analysis revealed that CTX‐1 exhibits a noteworthy binding affinity of −7.9 kcal/mol, challenging Erlotinib, a conventional lung cancer medication, which displayed a binding affinity of −7.3 kcal/mol. For a deeper understanding of CTX‐1's molecular mechanics, this study employed rigorous 100‐ns molecular dynamics simulations, demonstrating CTX‐1's remarkable stability in comparison with erlotinib. The Molecular Mechanics Poisson–Boltzmann Surface Area (MM‐PBSA) method further corroborated these results, with CTX‐1 showing a free binding energy of −105.976 ± 1.916 kJ/mol. The true prowess of CTX‐1 was tested against diverse lung cancer cell lines, including A549, Hop‐62 and H‐1299. CTX‐1 not only significantly outperformed erlotinib in anticancer activity but also exhibited a spectrum of therapeutic effects. It effectively diminished cancer cell viability, induced DNA damage, halted cell cycle progression, generated reactive oxygen species (ROS), impaired mitochondrial transmembrane potential, instigated apoptosis and successfully inhibited EGFR‐TKD. This study not only underscores the potential of CTX‐1 a formidable contender in lung cancer treatment but also marks a paradigm shift in oncological therapeutics, offering new horizons in the fight against this formidable disease.

## 1 INTRODUCTION

The epidermal growth factor receptor (EGFR), which comprises the external growth factor binding domain and the internal tyrosine kinase domain, is a key player in controlling cell growth through intricate signalling cascades^1^ The activation of EGFR, following ligand binding, instigates autophosphorylation through its innate tyrosine kinase activity, subsequently leading to a chain of vital cellular processes that determine cell behaviour and destiny.^2^, ^3^ Disruptions in downstream signalling, often due to EGFR mutations, are associated with the development of aggressive tumour traits, particularly seen in certain lung cancers. The introduction of tyrosine kinase inhibitors has significantly improved survival rates among patients with lung adenocarcinoma and mutant EGFRs.^4^, ^5^, ^6^

Despite recent progress in decoding the complex signalling networks that oversee cell survival, non‐small‐cell lung cancer (NSCLC) continues to present considerable challenges, with few curative options for advanced‐stage disease.^6^, ^7^ EGFR has been identified as a critical element in lung cancer development, with its overexpression noted in various human cancers, including NSCLC.^8^ EGFR tyrosine kinase mutations are frequently found in a subgroup of lung adenocarcinoma cases, underscoring their role in promoting cell proliferation.^9^ Although therapies targeting EGFR, such as tyrosine kinase inhibitors, have initially shown effectiveness, the rise of acquired resistance remains a significant barrier to the long‐term management of NSCLC. To address these issues, our study presents new perspectives on the therapeutic potential of 3,6‐diaminoacridine‐9‐carbonitrile (DAC) and its derivative CTX‐1 as viable treatments for lung cancer. Setting itself apart from preceding research, our study offers a detailed analysis of the binding affinity and stability of CTX‐1, contrasting it with the standard drug erlotinib. Additionally, we thoroughly investigated the anticancer effects of CTX‐1 on a variety of lung cancer cell lines. Our work further uncovers the intricate molecular mechanisms by which CTX‐1 operates, highlighting its impact on cancer cell viability, DNA damage induction, cell cycle arrest, generation of reactive oxygen species, disruption of mitochondrial transmembrane potential, apoptosis induction and EGFR‐TKD inhibition.

Significantly augmenting the existing body of knowledge, our study investigates the therapeutic potential of 3,6‐diaminoacridine‐9‐carbonitrile (DAC) a viable treatment options for lung cancer. Unlike previous research, which has primarily focused on other compounds, our study delves into the binding affinity and stability of CTX‐1, comparing it with the standard drug erlotinib. Furthermore, we offer a comprehensive evaluation of the biological anticancer effects of CTX‐1 across various lung cancer cell lines.

Going deeper, this research provides an understanding of the underlying molecular mechanisms through which CTX‐1 exerts its efficacy. This includes its influence on cancer cell viability, induction of DNA damage, cell cycle arrest, generation of reactive oxygen species, disruption of mitochondrial transmembrane potential, induction of apoptosis and inhibition of EGFR‐TKD. The novelty of this work lies in its comprehensive investigation of CTX‐1's therapeutic potential for lung cancer treatment, an area not extensively explored in prior studies. By illuminating the promising effects of CTX‐1 and offering a detailed understanding of its mode of action, our study introduces novel insights with the potential to significantly advance the field of lung cancer treatment.

## 2 MATERIALS AND METHODOLOGY

### 2.1 DFT calculations

The structural optimization of CTX‐1 and erlotinib was meticulously conducted using the advanced B3LYP/G method within the ORCA 4.1.2 program, aligning with methodologies parallel to Gaussian.^10^ A critical aspect of this process was the consideration of electronegative oxygen atoms, which proved vital in achieving precision in the optimisation outcomes. After optimisation, the focus shifted to the analysis of molecular frontier orbitals, encompassing both the highest occupied molecular orbital (HOMO) and the lowest unoccupied molecular orbital (LUMO). The determination of these orbital values was performed by applying vibrational frequency data derived from the Avogadro program. This particular software selection was instrumental because of its compatibility and seamless integration with the ORCA software, facilitating efficient visualisation of the results. The subsequent phase involved a thorough evaluation of the essential electronic properties. These included the HOMO–LUMO gap, which is a critical determinant of molecular reactivity and stability, along with the chemical potential, electronegativity, hardness (η) and softness (S). Each of these parameters was accurately calculated and represented, providing a comprehensive insight into the electronic characteristics of CTX‐1 and erlotinib. This robust and sophisticated methodology enabled not only the effective optimisation of CTX‐1 and erlotinib structures and facilitated a detailed assessment of their electronic features. More importantly, it sheds light on the key properties that underpin their potential bioactivity, thereby significantly contributing to the understanding of their therapeutic capabilities.

### 2.2 Molecular docking simulations

The crystal structure of EGFR‐TKD (PDB ID: 1 m17) reported by Stamos^11^ was retrieved from the RCSB Protein Data Bank for molecular docking analysis.^12^ Docking simulations were performed using PyRX software.^13^ Prior to the simulations, necessary preparations were conducted on the system, which involved removing water molecules, assigning partial charges, re‐optimizing the hydrogen bond network and minimizing the system using the default force field. The binding site for molecular docking was determined using Argus Lab Software.^14^ Specifically, the active site residues previously identified by Mir et al.,^15^ including Leu694, Ala719, Lys721, Glu738, Leu764, Thr766, Gln767, Leu768, Met769, Pro770, Phe771, Gly772, Leu820, Thr830 and Asp831, were selected as the target sites for docking simulations. The TKD was imported to the PyRx software and then made the protein as a macromolecule. The CTX‐1 and control molecule erlotinib were also imported to PyRx using the Open Babel module. The molecules were converted to the pdbqt format and minimised by using the mmff94 algorithm through the steepest method. Next, the grid was built around the binding site at centre X: 47.5642, Y: 39.0981, Z: 51.8301, with dimensions X: 19.511, Y: 18.5026 and Z: 22.7699 Å. The exhaustiveness score was set to 8, and the auto‐dock Vina algorithm was used for docking with random seed search. The interactions exhibited by the phytochemicals were identified by using Discovery Studio software.^16^

This rigorous molecular docking approach, employing the EGFR‐TKD crystal structure and advanced software tools, enables the assessment of ligand binding and facilitates the prediction of favourable binding conformations. It ensures a systematic exploration of ligand–protein interactions within the active site, providing valuable insights into potential binding modes and guiding further analysis and optimisation of the ligands.

### 2.3 Molecular dynamics simulations

Molecular dynamics simulations were conducted using Gromacs 22.04 software.^17^ The initial coordinates for the simulations were obtained from the docking simulations, and subsequent preparation steps were performed on the system. This involved removing water molecules and ligand atoms from the complex structure. The protein topology was generated using the AMBER99SB‐ILDN force field and the recommended TIP3P water model.^18^, ^19^ The ligand topology was generated using the acpype server and GAFF.^20^, ^21^ force field Both the ligand and protein topologies were placed in a three‐dimensional box.^22^ Subsequently, the system was solvated by adding 21,832 solvent molecules using the SPC module. Neutralization of the system was achieved by introducing a chloride ion (Cl^−^), and subsequent energy minimization was performed using the steepest descent method with 5000 steps. Further equilibration steps in the constant number of particles, pressure and temperature (NPT) and the constant number of particles, volume and temperature (NVT) ensembles were performed for 1 ns, employing a velocity‐rescaling thermostat and a modified Berendsen barostat.^23^, ^24^ The time step was set to 0.2 fs, the temperature was maintained at 300 K, and the pressure was set to 1 bar. Coulomb's cut‐off scheme of 1.2 nm was applied for the short‐range electrostatic interactions, whereas the long‐range electrostatic interactions were treated using the particle mesh Ewald algorithm (PME).^24^, ^25^, ^26^, ^27^ Each complex was subjected to 100‐ns simulations in the NPT ensemble, with temperature and pressure controlled using a velocity‐rescaling thermostat and a Parrinello–Rahman barostat. The resulting simulation trajectories were analysed using various Gromacs modules, including root mean square deviation (RMSD), root mean square fluctuation, SASA (solvent accessible surface area) and radius of gyration (Rg). In addition, principal component analysis (PCA) was employed to explore the motion of uncorrelated amino acid variables in the system.^28^, ^29^

The free energy binding (ΔG_bind_) of the CTX1‐ and erlotinib‐bound TKD complexes was determined using the g_mmpbsa tool.^30^, ^31^ The ΔG_bind_ values were calculated using all frames from the 100 ns simulations, and appropriate equations were applied for the estimation, as outlined in Kumari et al.^30^ The following equation was used to calculate the ΔG_bind_ (i).

(i) $$\;\Delta G_{\text{binding}}={\mathrm{\Delta G}}_{\text{complex}}-\left({\mathrm{\Delta G}}_{\text{protein}}\right)+\left({\mathrm{\Delta G}}_{\text{ligand}}\right)\ldots \ldots \ldots \ldots \ldots \ldots \ldots \ldots .$$

### 2.4 Chemicals

All chemicals were purchased from Sigma Aldrich and used without further purification.

### 2.5 Cell culture and reagents

All chemical reagents and mediums for cell culture were purchased from Sigma‐Aldrich. Lung cancer cell lines (A549, H‐1299 and HOP‐62) were obtained from the cell repository of the National Centre for Cell Science, Pune, Maharashtra, India. Dimethyl sulfoxide (DMSO) was used to prepare a stock solution (50 mM) of CTX‐1, which was then kept at −20°C until further use. In Dulbecco's Modified Eagle Medium (DMEM), which is supplemented with 10% foetal bovine serum (FBS) and penicillin and streptomycin antibiotics, the cells were grown at 37°C in a 5% CO2 and 95% humidity environment. For bioassay, cells were maintained and moved to the next passage for subculture.

### 2.6 In vitro cell proliferation assay

Three human lung cancer cell lines, namely A549, H‐1299 and HOP‐62, were cultured in 96‐well plates to evaluate the antiproliferative activity of CTX‐1 in comparison with the positive control Erlotinib, following a previously established protocol.^32^ For each well, 5 × 10^3^ cells were seeded in complete DMEM and incubated for 24 h. Subsequently, the cells were exposed to a range of concentrations (5–100 mM) of CTX‐1 and erlotinib for an additional 48‐h incubation period under 5% CO_2_ and humid conditions. After treatment, the cells were fixed using 30% trichloroacetic acid and stained with 0.4% sulforhodamine B (SRB). Unbound dye was removed by washing with 1% acetic acid, and the protein‐bound dye was extracted with 10 mM Tris. The absorbance of the plates was measured using SPECTRA max PLUS 384 microplate spectrophotometers. To determine the IC_50_ value, which represents the concentration required to inhibit 50% of cell proliferation, the Quest GraphTM IC_50_ Calculator online tool (AAT Bioquest, Inc., Sunnyvale, CA, https://www.aatbio.com/tools/ic50‐calculator) was employed. The experimental procedure was conducted in triplicate to ensure the robustness and reliability of the results.

### 2.7 Flow cytometry analysis of cell cycle progression

Flow cytometry analysis was performed to investigate the effect of CTX treatment on cell cycle progression in A549 cells. The cells were cultured in complete DMEM medium supplemented with 4.5 g/L glucose, 1% l‐glutamine, 10% FBS and 1% penicillin/streptomycin and maintained at 37°C with 5% CO_2_ in a humidified environment. Once the cells reached 80%–90% confluence, they were treated with the IC_50_ concentrations of CTX and erlotinib, which were diluted in 1% phosphate‐buffered saline (PBS). After 48 h of treatment, the cells were harvested, fixed with 70% ethanol and subsequently washed with 1X PBS. Staining was performed using propidium iodide (20 μg/mL) and 0.5% Triton‐X in 1X PBS, followed by the addition of 5 mg/mL RNase A. The samples were then incubated in the dark for 30 min. Flow cytometric analysis was conducted using a BD FACS Aria II flow cytometer to assess cell cycle progression and obtain relevant data.

### 2.8 Flow cytometry analysis for the apoptosis assay

For the determination of apoptotic activity in cancer cells, an apoptosis detection kit (Sigma‐Aldrich, St. Louis, MO) employing the Annexin‐V‐FITC method was used. In brief, a seeding density of 3 × 10^4^ A549 cancer cells per well was established in a 6‐well culture plate for experimental purposes, followed by a 24‐h incubation period. Subsequently, the cancer cells were treated with CTX‐1 and erlotinib at their respective IC_50_ concentrations. To label the cells, surface marker antibodies, including biotin‐conjugated Annexin V, FITC‐conjugated streptavidin and propidium iodide (PI), were employed. The trypsinized cells were suspended in a 1X binding buffer and then treated with Annexin V‐FITC conjugate in the dark at room temperature for 30 min. Data acquisition from a flow cytometer was performed using PI's 488‐nm excitation and 530‐nm emission wavelengths. By analysing the obtained data, the percentages of viable cells (Annexin V−/PI−), early apoptotic cells (Annexin V+/PI−) and late apoptotic/necrotic cells (Annexin V+/PI+) were acknowledged and calculated.

### 2.9 Assessment of nuclear morphology alterations induced by CTX‐1 and erlotinib in A549 lung cancer cells: A hoechst and propidium iodide staining study

Following a 48‐h treatment with CTX‐1 and erlotinib, cellular observations were conducted to assess alterations in nuclei morphology in A549 cells. To achieve this, A549 cells were seeded on poly‐L‐lysine‐coated coverslips in 6‐well plates and treated with the IC50 concentration of the drugs for 48 h. After the treatment period, the coverslips were carefully transferred to pre‐chilled methanol to fix the cells. Subsequently, the fixed cells were washed with 1X phosphate‐buffered saline (PBS) to remove residual fixative. The cells were then stained with a combination of 10 mM Hoechst and 15 mM propidium iodide (PI) solution at room temperature for 15 min, allowing for nuclear staining. Following staining, the coverslips were washed twice with PBS to remove excess dye. The stained cells were visualized using a Nikon Eclipse Ts2R‐FL inverted fluorescence microscope equipped with appropriate excitation filters. Excitation at a wavelength of 346 nm was used for Hoechst staining, whereas excitation at 460 nm was used for propidium iodide staining. The emitted fluorescence was observed and captured using suitable emission filters. During examination, cells that exhibited characteristic features such as nuclear condensation, membrane bleb formation, and the presence of apoptotic bodies were identified as undergoing apoptosis. This comprehensive cellular observation method allowed the evaluation of nuclear morphology changes, providing valuable insights into the apoptotic effects induced by CTX‐1 and erlotinib in A549 cells.

### 2.10 Assessment of mitochondrial membrane potential: effects of CTX‐1 and erlotinib on lung cancer cell lines (∆Ψm)

The effect of CTX‐1 and erlotinib on mitochondrial membrane potential was assessed using fluorescent dyes, namely rhodamine‐123 (Sigma‐Aldrich Co.; Ex/Em = 485 nm/535 nm) and DAPI (Sigma‐Aldrich Co.; Ex/Em = 358 nm/461 nm). Glass bottom plates were used to culture cells, which were then treated with CTX‐1 and erlotinib at their respective IC_50_ concentrations for 48 h. After treatment, cells were stained with rhodamine‐123 (15 μg/mL), JC‐1 (10 μg/mL) and DAPI (10 μg/mL) for 15 min at room temperature, followed by washing with PBS. Subsequently, cells were washed twice with 1X PBS, and fluorescence images were acquired at a magnification of 200× using an inverted fluorescent microscope (Nikon Eclipse Ts2R‐FL). For JC‐1 staining, untreated cells exhibited weak red fluorescence, indicating lower mitochondrial membrane potential, whereas treated cells displayed intense red fluorescence, indicating higher mitochondrial membrane potential. Similarly, untreated cells exhibited a relatively light blue DAPI stain with no significant morphological changes, whereas treated cells exhibited a prominent blue DAPI stain accompanied by altered morphological characteristics. The intensity of staining was quantified using ImageJ software. Furthermore, flow cytometry analysis was performed to detect changes in the mitochondrial transmembrane potential. Following 48 h of treatment with CTX‐1 and erlotinib, approximately 2 × 10^5^ cells were trypsinized, centrifuged at 3000 rpm for 5 min and washed with 1X PBS. Experimental and positive control cells were incubated with 1 μL of a 2.5 mg/mL JC‐1 fluorescent probe, and the cell suspension was vortexed and incubated for 15 min in darkness. The unbound probe was removed by centrifugation at 3000 rpm for 5 min at room temperature, and the cells were resuspended in 1000 μL of PBS for cytometric analysis. The cells were analysed using a BD‐FACS ARIA‐I flow cytometer with excitation from blue and yellow lasers at a detection wavelength of 585/42 nm.^33^

By employing these techniques, we gained valuable insights into the impact of CTX‐1 and erlotinib on mitochondrial membrane potential, providing a deeper understanding of their mechanisms of action in lung cancer cells.

### 2.11 Evaluation of intracellular reactive oxygen species levels induced by CTX‐1 and erlotinib in A549 lung cancer cells

Increased intracellular levels of reactive oxygen species (ROS) can have deleterious effects on cancer cells by disrupting vital cellular components, including organelles, lipid membranes and nucleic acids. To assess intracellular ROS levels, we employed the sensitive fluorescent probe 2′,7′‐dichlorofluorescein‐diacetate (DCFH‐DA), which undergoes oxidative conversion to generate the fluorescent compound 2′,7′‐dichlorofluorescein. A549 cells were seeded in 6‐well plates with cover glasses and treated with CTX‐1 and erlotinib for 48 h. Following the treatment period, the cells were carefully collected, washed twice with 1X phosphate‐buffered saline (PBS), and subsequently resuspended in a solution containing 10 mM DCFH‐DA (purchased from Molecular Probes Inc., Invitrogen). The cell suspension was then incubated for 30 min at room temperature in the dark to allow intracellular probe uptake and conversion. To visualise the labelled cells, fluorescence microscopy (Nikon Eclipse Ts2R‐FL) equipped with appropriate excitation filters was employed. Notably, the untreated control cells exhibited minimal fluorescence compared with the treated cells, indicating increased intracellular ROS levels upon treatment with both CTX‐1 and erlotinib. For flow cytometry analysis, a seeding density of 2 × 10^5^ A549 cells was used in 60‐mm Petri dishes, followed by a 24‐h incubation period. The cells were then treated with CTX‐1 and erlotinib for 48 h. Subsequently, the cells were trypsinized, washed with 1X PBS at 300 rpm for 3 min, and resuspended in 500 μL of 1X PBS. Intracellular ROS levels were assessed by adding 5 μL of H2DCFDA to the cell suspension and incubating it for 10 min. The unbound probe was removed by washing the cells with 1X PBS, and the resulting cell pellet was resuspended in 500 μL of 1X PBS. Flow cytometry analysis was performed using a BD‐FACS ARIA‐I flow cytometer with excitation/emission wavelengths set at 492–495/517–527 nm.^34^

### 2.12 Revealing the inhibitory effects of CTX‐1 and erlotinib on colony formation in lung cancer cells using a clonogenic assay

A clonogenic assay was performed to investigate the impact of CTX‐1 and erlotinib on colony formation. A total of 1000 cells were seeded in each well of a 6‐well plate and allowed to adhere overnight. Subsequently, the cells were treated with CTX‐1 and erlotinib for 48 h. Following the treatment period, the cells were allowed to grow undisturbed for 10–12 days to allow for colony formation. To visualise the colonies, the cells were fixed using 4% paraformaldehyde and stained with 0.5% crystal violet. The stained colonies were then counted for both the untreated and treated cell groups using Image‐J software (National Institute of Health, Bethesda, MD, USA).^35^ This software enabled accurate quantification of the number and size of colonies formed under each experimental condition.

The clonogenic assay provided valuable insights into the ability of CTX‐1 and erlotinib to inhibit the formation of colonies, which serves as an indicator of the cells' proliferative and clonogenic potential. By assessing colony formation, this assay offers a comprehensive evaluation of the long‐term effects of the treatments on cell survival and growth, providing valuable information about the efficacy of CTX‐1 and erlotinib in suppressing the clonogenic capacity of lung cancer cells.

### 2.13 Immunofluorescence imaging for the detection of EGF expression in cancer cells

A549 cells were cultured in six‐well plates at a density of 2 × 10^5^ cells/well and incubated at 37°C for 24 h. Following incubation, the media was aspirated, and the cells were washed with phosphate‐buffered saline (PBS). The cells were then treated with the IC50 concentration of the drug and incubated for 48 h. Subsequently, the cells were fixed with 3.7% paraformaldehyde for 10 min at room temperature. To ensure specific staining, the fixed cells were blocked with bovine serum albumin (BSA) in PBS at room temperature for 1 h, followed by additional washes. Next, the cells were incubated with a rabbit anti‐EGFR monoclonal antibody (10 μg/mL, Cetuximab clone C225; Merck, Billerica, Massachusetts, USA) for 2 h at room temperature. After three washes to remove unbound antibodies, the cells were further incubated with goat anti‐rabbit IgG conjugated to fluorescein isothiocyanate (FITC) (1:50 dilution in PBS containing BSA at 0.2 mg/mL; Santa Cruz Biotechnology, Santa Cruz, CA, USA) for 1 h at room temperature. Following another round of washes, cells were stained with Hoechst 33342, a fluorescent DNA dye, to visualise cell nuclei. The stained cells were observed under a fluorescence microscope (Carl Zeiss) to examine the localization and expression of EGFR.

This detailed experimental protocol, adapted from,^36^ enabled the investigation of EGFR expression and localization in A549 cells upon treatment with the IC_50_ concentration of the drug. The specific staining and imaging techniques provided valuable insights into the interaction between the drug and the EGFR protein within the cellular context.

## 3 RESULTS AND ANALYSIS

### 3.1 Density functional theory (DFT) analysis

DFT stands as a cornerstone in computational chemistry for probing the electronic structure of molecules, offering a window into their chemical properties and reactivity. The detailed image provided is paired with the insightful narrative and encapsulates the power of DFT in unravelling the molecular intricacies of compounds such as CTX‐1 and erlotinib. The analysis draws particular attention to the HOMO and LUMO energies, which are pivotal in predicting how a molecule will donate or accept electrons—key factors in determining their reactivity and interaction with biological systems. In the realm of molecular stability, the HOMO–LUMO gap emerges as a pivotal indicator, where CTX‐1 showcases a narrower gap, suggestive of enhanced stability relative to erlotinib, which bears a wider gap indicative of higher reactivity. This is more than an academic exercise; it carries implications for how these molecules may behave in physiological environments, with CTX‐1 displaying a pronounced propensity to maintain its integrity, suggesting less reactivity and potentially greater predictability in its interactions within the body.

The DFT calculations extend beyond just energy levels, providing a suite of chemical descriptors—chemical potential, electronegativity, hardness and softness each painting a detailed portrait of the molecular character. Erlotinib, for instance, is quantified with a hardness of 1.96 eV and a softness of 0.50 eV, whereas CTX‐1 is characterised by a hardness of 1.33 eV and a softness of 0.74 eV (Figure S1, Table S1 and Raw R1 & R2 in Data S1). This robust DFT‐based analysis hence provides a compelling narrative of CTX‐1's chemical prowess, bolstered by quantitative data and visual evidence, making a strong case for its continued investigation as a molecular entity in cancer treatment.

### 3.2 Molecular docking reveals superior binding affinity and interactions of CTX‐1 with EGFR‐TKD compared with erlotinib

Molecular docking analyses were conducted to investigate the interactions between CTX‐1 and EGFR‐TKD. Initially, the docking method was validated by redocking the co‐crystallized ligand (erlotinib) in the binding site, and the calculated RMSD was found to be 1.5 Angstroms. The conformations of the native ligand and the redocked co‐crystallised ligand are shown in Figure S2.

**FIGURE 1:**
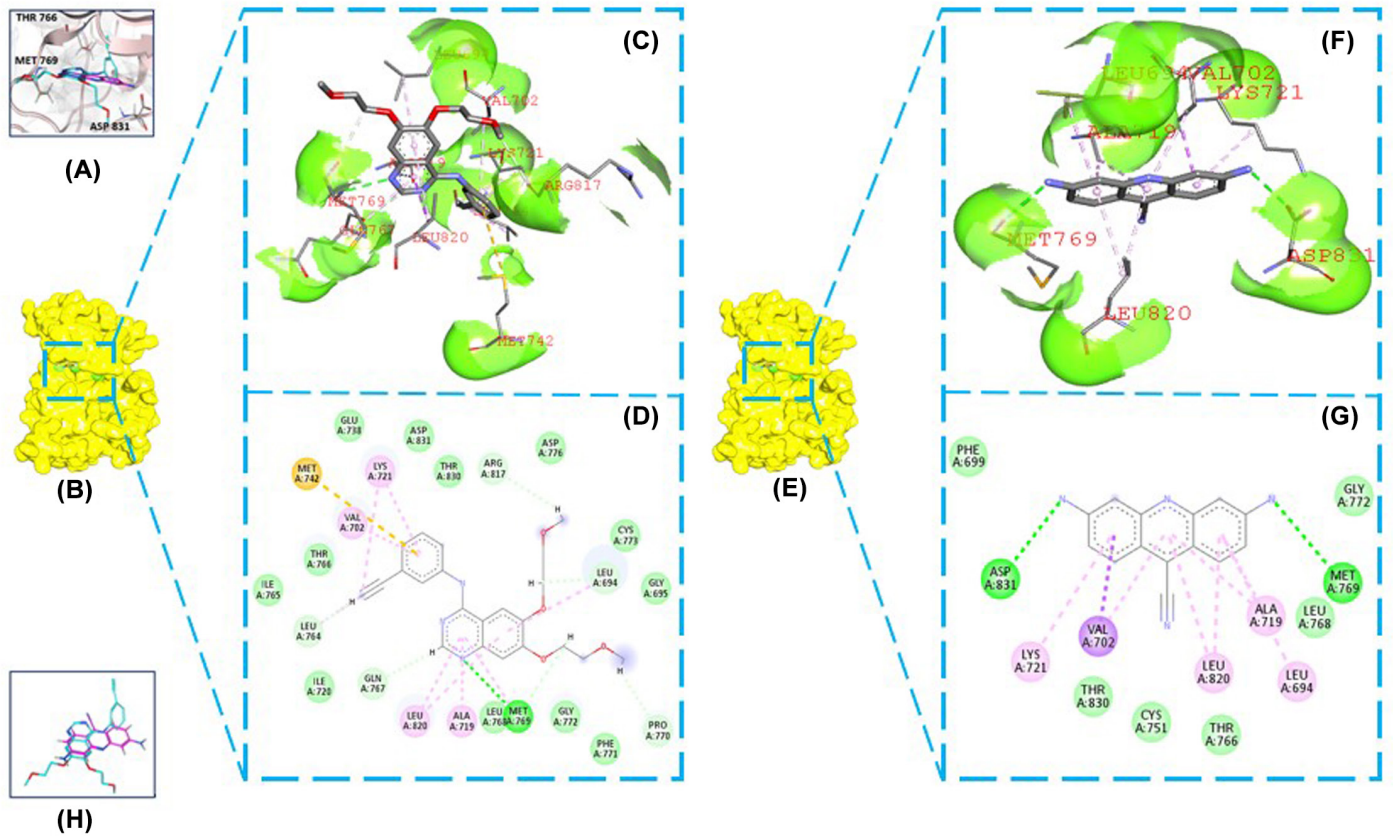
(A) Molecular docking interactions of CTX‐1 and erlotinib with TKD; (B) Complex of erlotinib and TKD, (C) 3D interactions of erlotinib with TKD (D) 2D interactions of erlotinib with TKD. (E) TKD complexed with CTX‐1 (F) 3D interactions of the CTX‐1 with TKD, (G) 2D interactions of CTX‐1 with TKD and (H) binding orientations of CTX‐1 and erlotinib in the binding site.

For comparative analysis, the reference molecule erlotinib was included. The molecular docking simulations revealed that CTX‐1 exhibited a superior binding affinity with TKD, with a binding affinity score of −7.9 kcal/mol, whereas erlotinib demonstrated a binding affinity score of −7.3 kcal/mol. The docking interactions of CTX‐1 with EGFR‐TKD involved a conventional hydrogen bond interaction with MET769 and ASP831, and the pi‐sigma contact was found with the amino acid VAL702. Other interactions, such as pi‐alkyl interactions were observed with amino acids LEU694, ALA719, LYS721 and LEU820 (Figure 1F,G). In contrast, the reference molecule erlotinib showed interactions with MET769 through conventional hydrogen bonding. In addition, pi‐sigma interactions were observed with amino acids LEU694 and LEU820 and pi‐sigma interactions with ALA719, LYS721 and LEU764 (Figure 1C,D; Raw R3 in Data S1).

These docking analyses provide insights into the specific interactions between CTX‐1 and EGFR‐TKD, highlighting the potential binding modes and molecular interactions that contribute to their binding affinity (Figure 1A,H). The observed interactions suggest that CTX‐1 may effectively target the EGFR‐TKD protein, offering promising prospects for its role as a potential treatment option for lung cancer.

### 3.3 Molecular dynamics simulations

Molecular dynamics simulations provide valuable insights into the dynamic behaviour of ligand‐bound targets and are widely used in both the industrial and academic sectors. In this study, we employed molecular dynamics simulations to investigate the movements and conformational stability of CTX‐1 and erlotinib over a simulation period of 100 ns. The conformations of both the ligands and the protein were monitored throughout the simulations, and the root‐mean‐square deviation (RMSD) was calculated to assess their stability.

**FIGURE 2:**
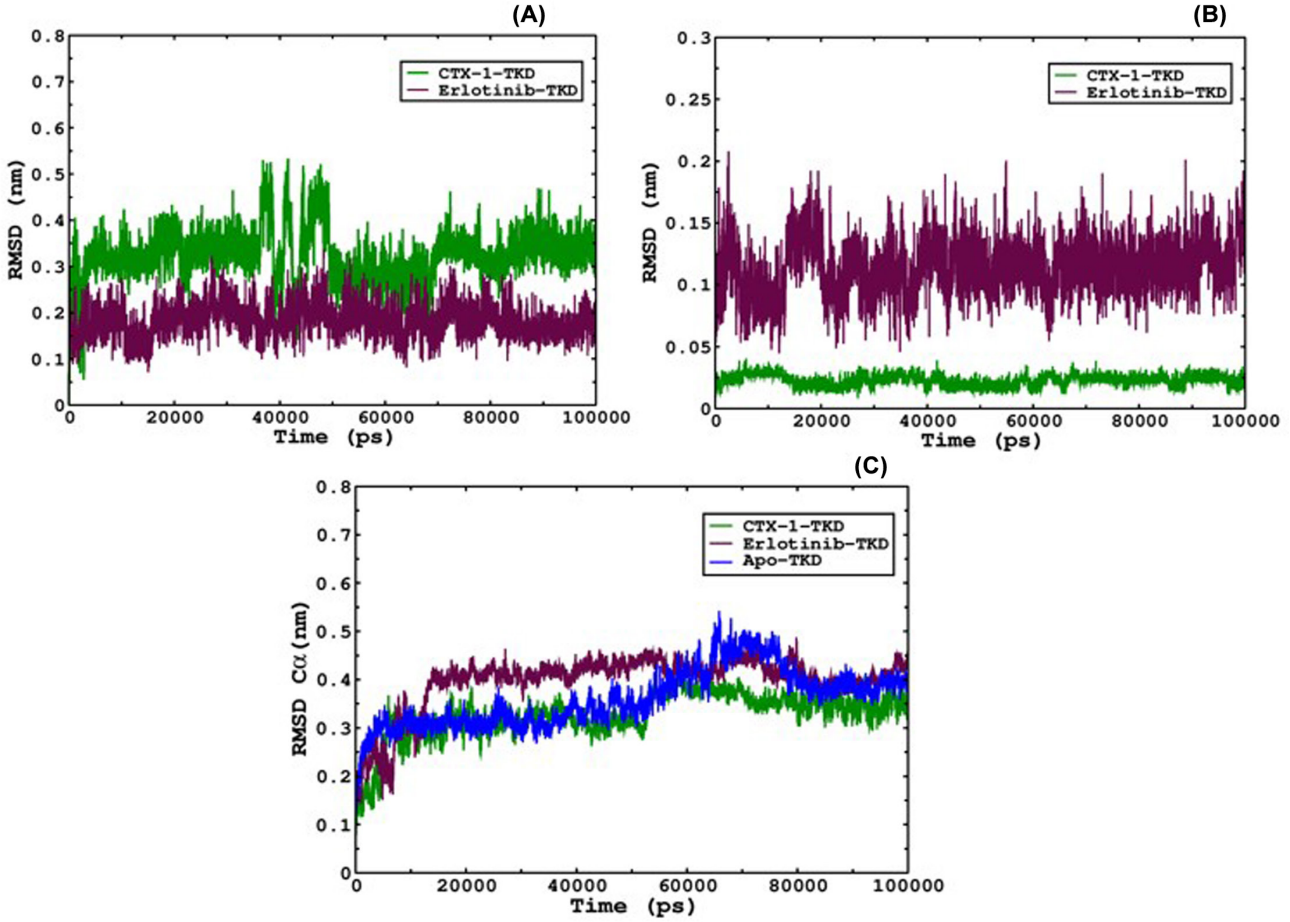
(A) The conformations of CTX‐1 were continuously monitored by superposing them onto the TKD protein structure over a 100‐ns simulation in an aqueous medium. The erlotinib trajectory concerning TKD was obtained from our own previously reported data^12^, ^29^ only to show the comparative analysis of erlotinib and CTX‐1. Both compounds exhibited remarkable stability throughout the simulation period. The RMSD trajectories of CTX‐1 and erlotinib are depicted in green and maroon colours, respectively. (B) The RMSD profiles of CTX‐1 and erlotinib were analysed by superposing them onto their respective ligand structures throughout the simulations. The resulting trajectories of both ligands are presented in the figure, providing insights into their dynamic behaviour and structural fluctuations over time. (C) The Cα RMSD of both complexes, namely CTX‐1‐bound TKD and erlotinib‐bound TKD, was thoroughly investigated. The analysis revealed that the CTX‐1‐bound TKD exhibited greater stability compared with the erlotinib‐bound TKD, and the Apo complex represented as blue, as evidenced by the lower RMSD values observed for CTX‐1.

Root mean square deviation analysis revealed that both CTX‐1 and erlotinib exhibited relatively stable binding to the cytosolic domain tyrosine kinase of the epidermal growth factor receptor (EGFR) throughout the simulations. The RMSD values for CTX‐1 and erlotinib ranged between 3 Å, indicating that CTX‐1 maintained stability comparable to that of erlotinib within the binding site (Figure 2A). Furthermore, the conformations of CTX‐1 and erlotinib were compared by superimposing them on their respective ligands. The RMSD values for erlotinib were found to be around 2 Å, while CTX‐1 demonstrated even greater conformational stability with RMSD values within the 0.5 Å range (Figure 2B).

To assess the overall stability of the complex, the movements of the Cα atoms of the tyrosine kinase domain (TKD) bound to CTX‐1 and erlotinib were analysed. The RMSD values serve as an indicator of the complex's stability, with higher RMSD values suggesting lower stability and vice versa. The erlotinib‐bound TKD complex reached equilibrium after approximately 15 ns of simulation, with RMSD values ranging from 3.8 to 4.2 Å. In contrast, the CTX‐1‐bound TKD complex achieved equilibrium within 5 ns of simulation, and the RMSD remained within the range of 2.8 to 4 Å throughout the simulation period. The RMSD of the Apo complex was found to be almost similar to that of the EGFR‐TKD complex with CTX‐1. These results indicate that the CTX‐1‐bound TKD complex exhibited greater stability than the erlotinib‐bound TKD complex (Figure 2C; Raw R4, R5 & R6 in Data S1).

The overall RMSD analysis provided a comprehensive understanding of the structural dynamics and stability of CTX‐1 and erlotinib during molecular dynamics simulations (Figure 2). Superposition of conformations and examination of RMSD trajectories revealed the behaviour and deviations of these compounds, demonstrating the stability of both CTX‐1 and erlotinib throughout the simulation. Moreover, comparing Cα RMSD values highlights the superior stability of the CTX‐1‐bound TKD complex compared with that of the erlotinib‐bound TKD complex. These findings deepen our understanding of the molecular interactions and stability of these compounds, supporting their potential as effective treatments for lung cancer. By elucidating the conformational movements and stability of CTX‐1 and erlotinib within the EGFR TKD binding site, this study provides valuable insights into the dynamics of these compounds and their potential as targeted therapies for lung cancer.

**FIGURE 3:**
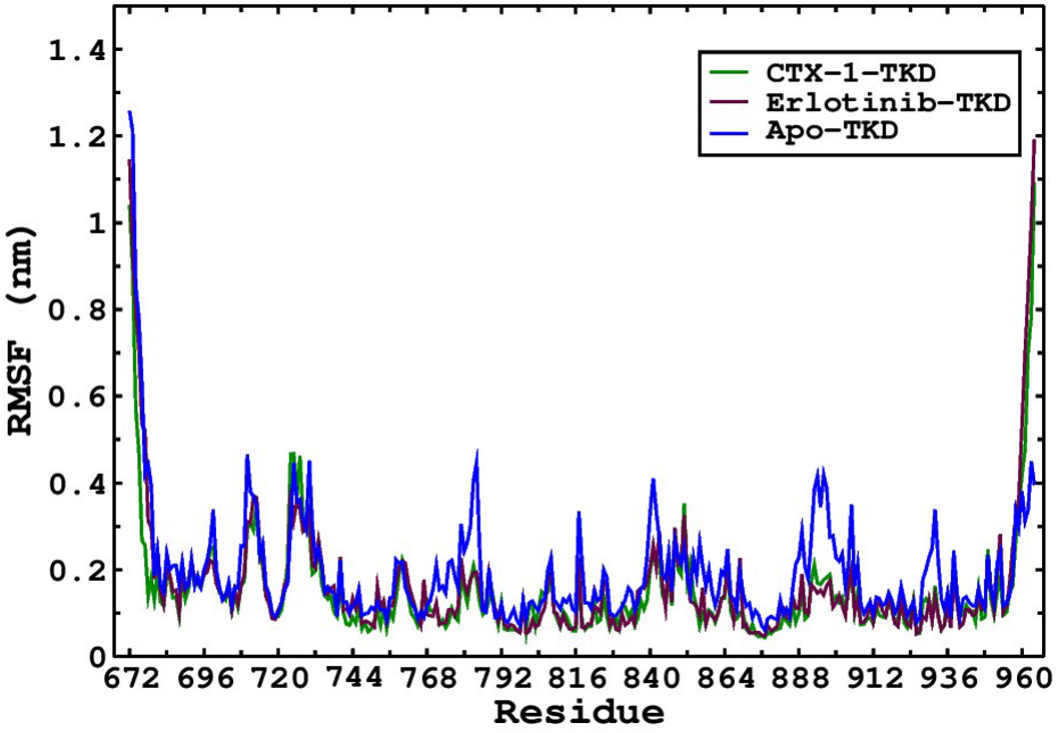
Fluctuation among the whole sequence of the TKD bound with CTX‐1, erlotinib and Apo forms. Higher fluctuations were observed in the binding site of the Apo TKD protein than in TKD bound with CTX‐1 and erlotinib.

Furthermore, we conducted a detailed analysis of the dynamic behaviour of the protein–ligand complexes by examining the root mean square fluctuation (RMSF) using the RMSF module of the Gromacs software. The RMSF analysis provides valuable insights into the fluctuations of individual amino acids throughout the simulation, allowing us to understand the conformational dynamics of the complexes. The results revealed distinct patterns of fluctuations within the protein structure. Specifically, higher fluctuations were observed at the terminal ends of the protein in both the CTX‐1‐EGFR‐TKD and erlotinib‐EGFR‐TKD complexes, indicating greater flexibility in these regions. Conversely, the amino acids located in the binding site exhibited relatively fewer fluctuations, suggesting a more stable interaction between the ligands and the receptor. In particular, the binding site amino acids displayed fluctuations within a narrow range of 0.05–0.22 nm (Figure 3). This finding indicates that the residues directly involved in the binding interaction maintain a relatively stable conformation throughout the simulation, supporting the robustness of the ligand–protein interactions in both the CTX‐1‐EGFR‐TKD and erlotinib‐EGFR‐TKD complexes.

The detailed analysis of RMSF, as illustrated in Figure 3, provides a comprehensive understanding of the dynamic behaviour of the protein–ligand complexes. The RMSF of the Free EGFR‐TKD was found to be higher specifically in the binding site in comparison to complexes with erlotinib and CTX‐1. By identifying the regions of higher flexibility and those involved in stable binding interactions, we gain valuable insights into the conformational stability of the complexes. These findings further support the efficacy of CTX‐1 as a potential treatment for lung cancer and provide a deeper understanding of its interaction with EGFR‐TKD. (S4, S5 & S6 in Data S1).

In addition to assessing the binding affinity and stability of CTX‐1 and erlotinib with EGFR‐TKD, we further investigated the compactness of the complexes by calculating the gyration radius. The radius of gyration is a measure of the overall size and compactness of the protein structure. A higher radius of gyration indicates compactness of the protein; if the protein is stable, it will likely maintain a relatively steady value of Rg. To determine the compactness of the TKD bound with CTX‐1 and erlotinib, we performed a comparative analysis by taking free TKD as a reference. The relatively steady values of Rg decreased; hence, the Apo protein unfolded over simulation, but the TKD bound with CTX‐1 and erlotinib showed relatively steady Rg values and were compact. The radius of gyration values were calculated at the end of the molecular dynamic's simulations. Remarkably, both complexes exhibited similar levels of compactness, as evidenced by the radius of gyration value ranging from 2.01 to 1.96 nm after the simulations (Figure S3). (S4, S5 & S6 in Data S1).

The radius of gyration indicates that both CTX‐1 and erlotinib form stable and compact complexes with the EGFR‐TKD protein. The comparable compactness suggests that both compounds can effectively interact with the target protein and potentially exert their biological effects. These findings provide additional insights into the structural characteristics of CTX‐1 and erlotinib complexes, supporting their potential as therapeutic agents for lung cancer treatment. Moreover, to assess protein folding during the molecular dynamics simulations, the solvent accessible surface area (SASA) was calculated. The SASA value provides insights into the degree of protein folding, with higher values indicating more stable complex formation and vice versa. In this study, both the CTX‐1‐EGFR‐TKD and erlotinib‐EGFR‐TKD complexes exhibited parallel protein folding throughout the simulations. The calculated SASA value for both complexes ranged from 152.5 to 162.5 nm^2^.These results demonstrate that higher protein folding was observed in CTX‐1‐ and erlotinib‐bound TKD than in free TKD (Raw R4, R5 & R6 in Data S1).

Principal component analysis (PCA) was employed to gain comprehensive insights into the structural dynamics and clustering patterns exhibited by the studied complexes. PCA is a widely recognised technique that reduces the dimensionality of complex data while retaining the most informative features. In this study, PCA was applied to the trajectory data obtained from the molecular dynamic's simulations of CTX‐1‐EGFR‐TKD and erlotinib‐EGFR‐TKD complexes. The analysis focused on capturing the fluctuations and correlations of the atomic positions over the simulation time. The PCA results revealed distinct clusters that corresponded to different conformational states or ensembles of the complexes. These clusters provide valuable information about the major conformations explored by the complexes during the simulations, offering insights into their dynamic behaviour.

**FIGURE 4:**
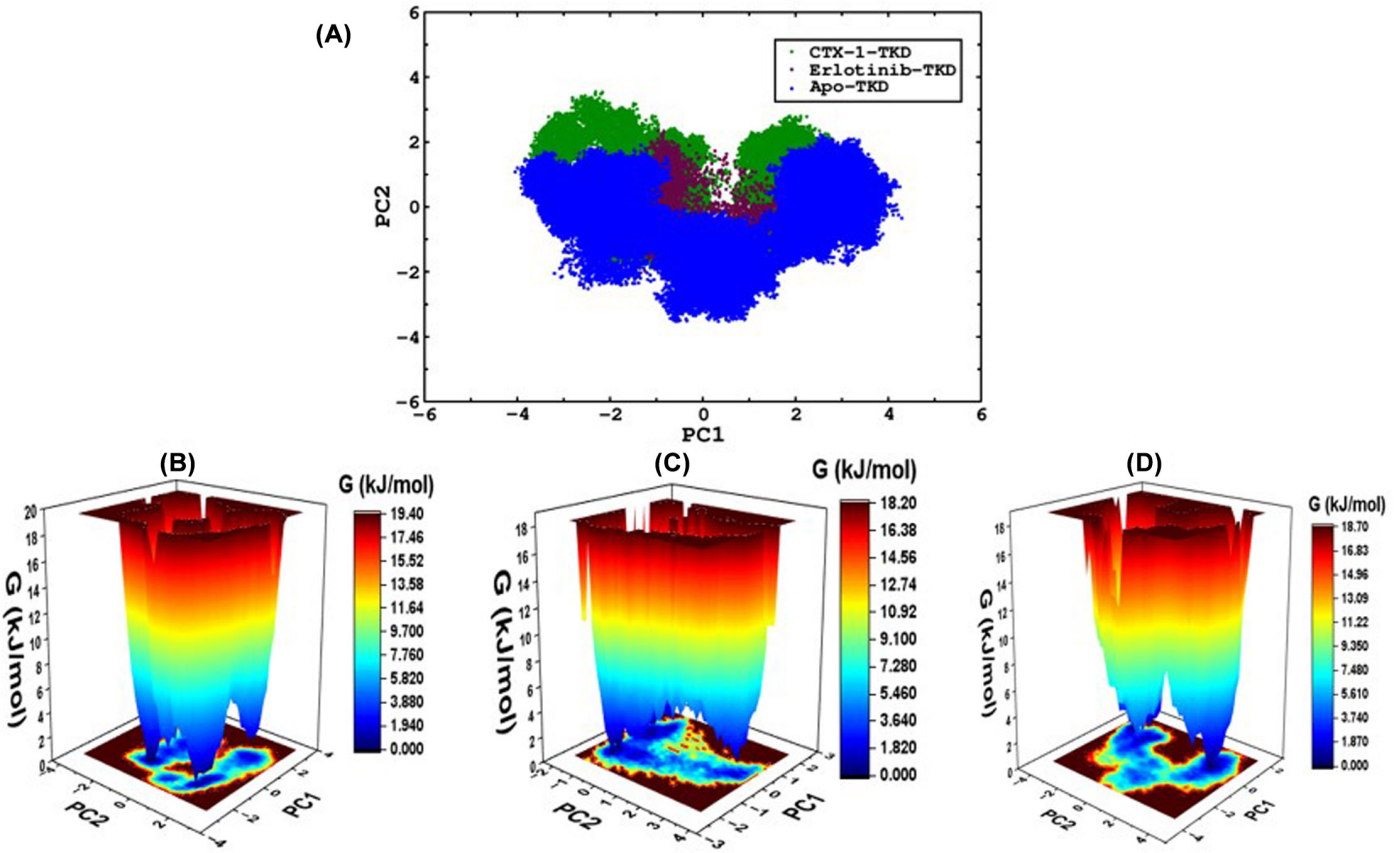
(A) PCA was calculated to determine the clustering of both complexes with CTX‐1 and erlotinib (dark green and maroon) and free EGFR‐TKD, represented in blue. The free energy landscape conformational plots for CTX‐1 complexed with TKD (B). Erlotinib‐bound TKD (C) and Apo protein (D).

Clustering analysis facilitated the identification of coherent groups that represent structurally similar conformations within complex ensembles. This characterization of distinct clusters allowed for a deeper understanding of the conformational diversity and potential functional states of the complexes. Furthermore, PCA analysis provided a visualization of the principal components, enabling the observation of concerted movements and correlations among key residues and domains within the complexes. This information provided valuable insights into the collective motions and interplay of different regions within the CTX‐1‐EGFR‐TKD and erlotinib‐EGFR‐TKD complexes (Figure 4A).

Overall, PCA analysis significantly contributed to our understanding of the structural dynamics and clustering patterns exhibited by the complexes. This comprehensive analysis offers important insights into their conformational diversity, potential functional states and collective motions, further reinforcing the findings obtained from binding affinity calculations, molecular dynamics simulations and other experimental evaluations. The free energy landscapes (FEL) were obtained from PCA to explore the conformational changes exhibited by the protein in complex with the ligand and free EGFR. The FEL was obtained by using the g_sham module of the Romans, and the more stable conformations were represented in dark green; the cyan, green and yellow colours represented the stable conformations, whereas the red colour represented the less stable conformations. The higher FEL exhibited by CTX‐TKD (Figure 4B), erlotinib‐TKD (Figure 4C), and APO (Figure 4D) was found to be 19.40, 18.20 and 18.70 kJ/mol, respectively. These results corroborate that each complex including free protein showed comparable stable conformations and was within permissible limits (Raw R4, R5 and R6 in Data S1).

### 3.4 Free energy calculations

The free binding energy was calculated using the Molecular Mechanics Poisson– Boltzmann Surface Area (MM‐PBSA) method, as outlined in Kumari et al.^30^ The binding energies were determined for CTX‐1 and the reference molecule erlotinib when bound to the TKD. Our analysis revealed that CTX‐1 exhibited a ΔG_bind_ of −105.976 ± 1.916 kJ/mol, whereas erlotinib displayed a ΔG_bind_ of −130.595 ± 0.098 kJ/mol.^12^, ^29^

Furthermore, we investigated the hydrophilic and hydrophobic interaction energies of CTX‐1 with our own previously reported energy of erlotinib.^12^, ^29^ The hydrophilic interaction energy for CTX‐1 was found to be −121.048 ± 0.901 kJ/mol, whereas Erlotinib exhibited a hydrophilic interaction energy of −222.066 ± 0.532 kJ/mol. In addition, the hydrophobic interactions observed for CTX‐1 and erlotinib were − 78.117 ± 2.474 kJ/mol and − 53.449 ± 0.632 kJ/mol, respectively.

The analysis of free energy binding strongly suggests that CTX‐1 acts as a potent inhibitor of EGFR TKD. This conclusion is supported by the ΔG_bind_ values of both molecules, which exceed 100 kJ/mol **(**Table 1 **).** These findings indicate that CTX‐1 can effectively inhibit the activity of EGFR TKD and further support its role as a promising candidate for lung cancer treatment **(**Raw R4 and R5 in Data S1 **).**

**TABLE 1:** Free binding energies of CTX‐1 and erlotinib and their associated parameters.

Complex	ΔE_VdW_	ΔE_Elec_	ΔE_PSol_	ΔE_SASA_	ΔG_binding_
CTX‐1‐TKD	−121.048 ± 0.901	−78.117 ± 2.474	106.983 ± 1.394	−13.781 ± 0.073	−105.976 ± 1.916
Erlotinib‐TKD	−222.066 ± 0.532	−53.449 ± 0.632	165.597 ± 0.752	−20.680 ± 0.042	−130.595 ± 0.098

### 3.5 Cellular‐Level evaluation of the antiproliferative activities of CTX‐1 and Erlotinib in human lung cancer cell lines

**FIGURE 5:**
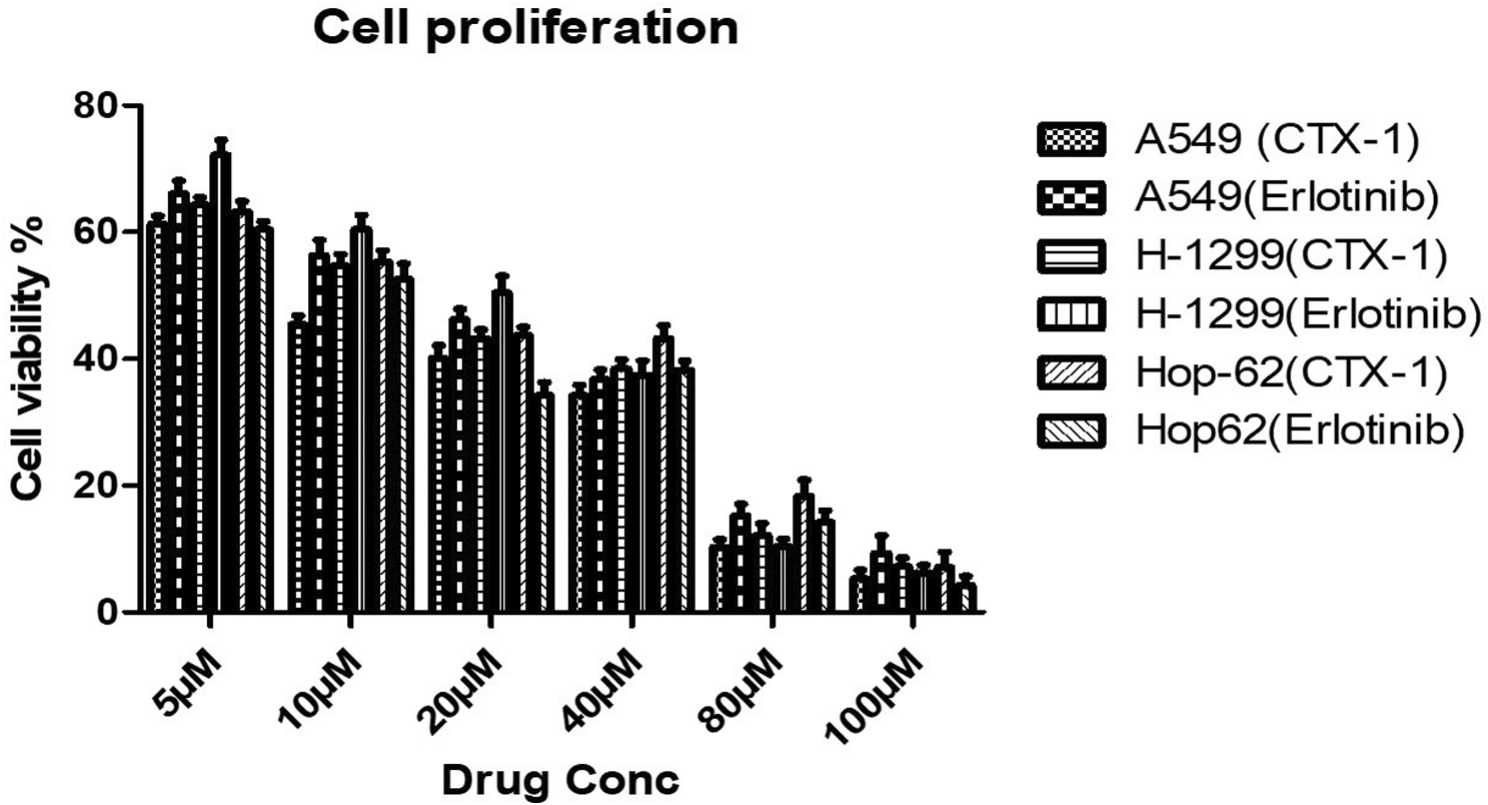
Inhibition of the growth of human lung cancer cells (A549, H‐1299 and HOP‐62) is more effectively achieved by CTX‐1 and erlotinib compared with the control group over a 72‐h period. (Each data point represents the average result from three independent experiments.)

Building upon our in silico findings, we conducted rigorous cellular‐level investigations to assess the impact of CTX‐1 and erlotinib on the growth of lung cancer cells. Specifically, we examined the antiproliferative activities of these compounds in three distinct human lung cancer cell lines, namely A549, H‐1299 and HOP‐62. By determining the IC50 values for CTX‐1 and erlotinib in these cell lines, we obtained valuable insights into their respective potency and efficacy. The compiled IC_50_ values are graphically depicted in Figure 5 and underscore the variability observed among the different lung cancer cell types, indicating a cell type‐dependent response to the compounds. In evaluating the inhibitory effects of CTX‐1 and erlotinib on different cancer cell lines, compelling findings have emerged. When CTX‐1 was used against the A549 cancer cell line, the IC_50_ value was determined to be 7.3 ± 0.5 μM. On the other hand, erlotinib exhibited an IC_50_ value of 12.2 ± 0.3 μM on the same cell line.

Furthermore, the application of CTX‐1 to the H‐1299 cell line resulted in an IC_50_ value of 13.6 ± 0.7 μM, while erlotinib displayed an IC_50_ value of 11.7 ± 0.4 μM. On evaluating the effect against the Hop‐6 cell line, the IC_50_ value for CTX‐1 was found to be 8.5 ± 1.2 μM, compared with erlotinib, which displayed an IC_50_ value of 7.3 ± 0.5 μM. These findings provide intriguing insights into the inhibitory potency of CTX‐1 and erlotinib against these specific cancer cell lines.^37^, ^38^

While definitive conclusions regarding the susceptibility of cancer cells to CTX‐1 and erlotinib cannot be drawn solely based on these findings, it is evident that the mechanisms of cell death and the inhibition of EGFR represent potential targets for the therapeutic effects of these drugs. Further investigations are warranted to elucidate the specific molecular pathways and signalling cascades affected by CTX‐1 and erlotinib, providing deeper insights into their antiproliferative effects in lung cancer cells. Such investigations will contribute to our understanding of the underlying mechanisms and potentially pave the way for the development of novel therapeutic strategies targeting lung cancer.

### 3.6 CTX and erlotinib induced apoptosis of cancer cells

To determine if CTX induces apoptotic cell death similar to that induced by erlotinib in lung cancer cells, we conducted Annexin V/propidium iodide (PI)‐based apoptosis assays. During apoptosis, phosphatidylserine, typically located on the inner leaflet of the cell membrane, translocates to the outer leaflet, which can be fluorescently assessed through annexin V binding, a characteristic biochemical event of the apoptotic process. Propidium iodide, a fluorescent DNA‐binding dye that cannot penetrate live cells, can only enter cells during the later stages of apoptosis when membrane integrity is compromised. Using flow cytometry analysis, we quantified the population of apoptotic cells.

**FIGURE 6:**
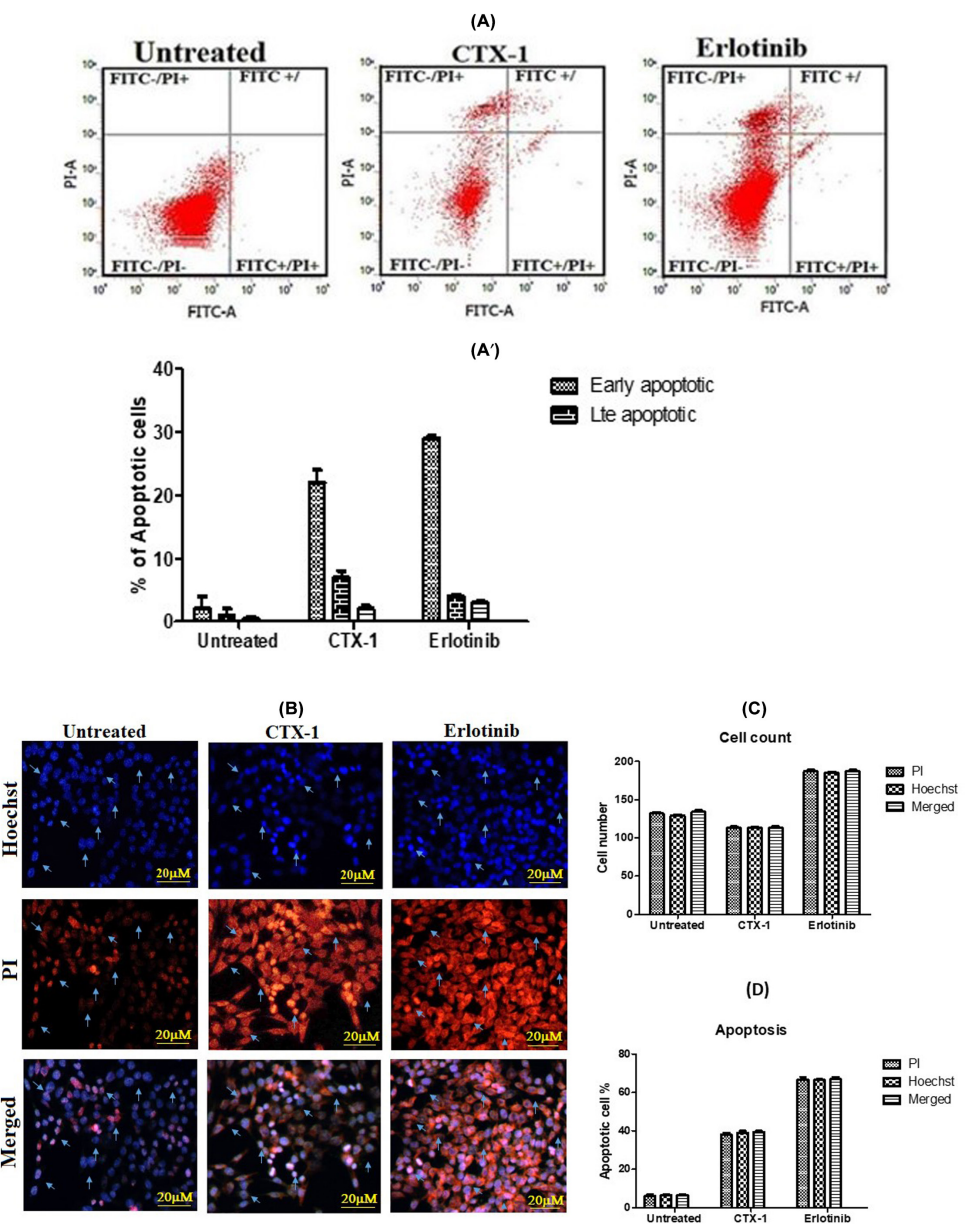
(A) Flow cytometry analysis of phosphatidylserine (PS) exposure in A549 cells treated with CTX‐1 and erlotinib at their respective IC_50_ concentrations for 48 h, compared with untreated control cells. Annexin‐V and propidium iodide (PI) staining were used to distinguish three subpopulations: PI− and Annexin V− cells represent viable cells with intact membrane and preserved amino‐phospholipid asymmetry; PI− and Annexin V+ cells represent early apoptotic cells with intact cellular membrane exposing phosphatidylserine, and PI+ and Annexin V+ cells represent late apoptotic cells with compromised asymmetry and membrane permeability (A'). (B) Morphological changes associated with apoptosis, such as chromatin condensation, membrane blebbing and formation of apoptotic bodies, were visualised using fluorescence microscopy. Panels show representative images of cells stained with PI, Hoechst and merged PI and Hoechst channels for untreated (left panels) and cells treated with IC_50_ concentrations of CTX‐1 and erlotinib (right panels) for 48 h. Apoptotic cells resulting from 48 h of drug treatment are visible; (C) Graphical representation of total counted cells and (D) The percentage of the apoptotic cells shown by CTX‐1 and erlotinib is plotted and compared with the untreated cells.

Following a 48‐h treatment of lung cancer cell lines with CTX and erlotinib at their respective IC_50_ concentrations, the percentages of early and late apoptotic cells were determined and compiled. Representative flow cytometry figures after CTX and erlotinib treatments are shown in Figure 6A–C. The control untreated cell culture, after 48 h of incubation, exhibited minimal levels of early (3.5%) and late (1.0%) apoptotic cells, which were considered to be the background level of cell death resulting from routine stress during cell culture. In contrast, treatment with CTX‐1 and erlotinib led to significantly higher percentages of early and late apoptotic cells compared with the untreated control. Specifically, these percentages were 15%, 45%, 56% and 5%, respectively (Raw R7 in Data S1).

These results provide evidence that both CTX‐1 and erlotinib induce apoptotic cell death in lung cancer cells, demonstrating their potential as effective agents in promoting apoptosis. The observed increase in apoptotic cell populations following treatment with CTX‐1 and erlotinib highlights their ability to target and trigger programmed cell death pathways, further supporting their potential as promising therapeutic options for lung cancer.

### 3.7 Effects of CTX‐1 and erlotinib on the cell cycle profile of A549 cells: induction of G2/M phase arrest

**FIGURE 7:**
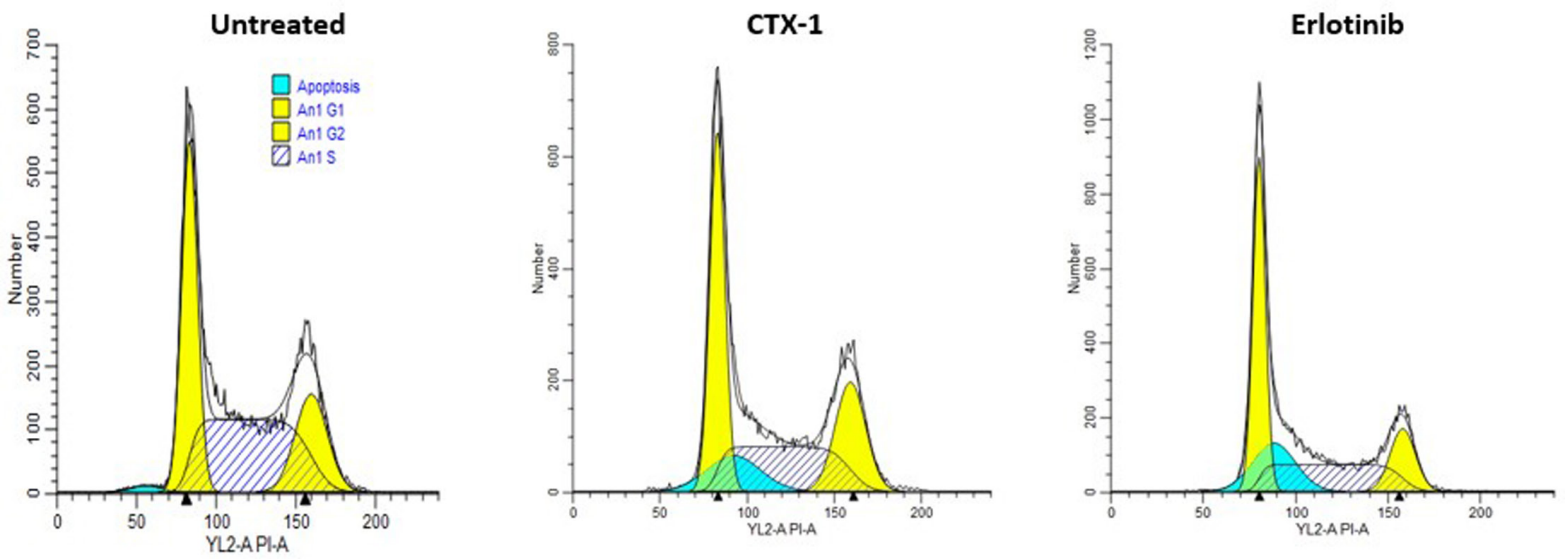
Disruption of G2/M phase cell cycle progression by CTX‐1 and erlotinib: Induction of hypodiploid (sub‐G1) DNA peak, indicative of apoptotic cells.

We performed an in‐depth analysis of the effects of CTX‐1 and erlotinib at their respective IC_50_ concentrations on the cell cycle profile of A549 cells using flow cytometry, as depicted in Figure 7. Examination of the cell cycle profile provides valuable insights into the induction of cell death by the tested compounds. The accumulation of fluorescently labelled DNA serves as a reliable indicator of cell cycle distribution and the occurrence of cell death events. In the cell cycle, the G1 phase is characterised by cells with 2 N DNA content, whereas the G2 and M phases consist of cells with a doubled 4 N DNA content. During DNA replication, the S phase is represented by cells with both 2 N and 4 N peaks. Cells undergoing various stages of DNA degradation, indicative of dying cells, exhibit DNA content below the 2 N threshold.

The impact of CTX‐1 and erlotinib on cell cycle progression was evaluated by flow cytometry in A549 cells treated with their respective IC_50_ concentrations for 48 h. The flow cytometry panels depict the assessment of cell cycle profiles. Treatment with CTX‐1 and erlotinib resulted in the disruption of G2/M phase cell cycle progression, as evidenced by a significant increase in the hypodiploid (sub‐G1) DNA peak. The appearance of this peak signifies the presence of apoptotic cells undergoing DNA degradation.

These findings indicate that both CTX‐1 and erlotinib induce cell cycle arrest and promote apoptosis in A549 cells. The detailed analysis of cell cycle progression provides valuable insights into the mechanisms of action of these compounds and their potential as therapeutic agents for lung cancer treatment. A significant alteration in the cell cycle profile following treatment with CTX‐1 and erlotinib at their respective IC_50_ concentrations for 48 h was observed. FACS analysis demonstrated a substantial increase in the number of cells in the G2/M phase compared with untreated cells, indicating cell cycle arrest. This observation suggests that both CTX‐1 and erlotinib induce cell cycle perturbations, potentially leading to cell death in A549 cells. The detailed cell cycle analysis provides further evidence of the efficacy of these compounds in altering the proliferative behaviour of lung cancer cells, supporting their potential as therapeutic agents for lung cancer treatment (Raw R8 in Data S1).

### 3.8 Disruption of mitochondrial membrane potential (∆Ψm) by CTX‐1 and erlotinib: insights into apoptotic pathways in lung cancer cells

Disruption of mitochondrial function is a distinctive characteristic of programmed cell death during its early stages. This perturbation affects both the oxidation–reduction potential and membrane potential of mitochondria. The opening of the mitochondrial permeability transition pore (MPTP), which facilitates the transport of ions and small molecules, is believed to be associated with changes in the membrane potential. Subsequently, the equilibration of ions occurs due to the uncoupling of the respiratory chain, resulting in the release of cytochrome C into the cytosol. To assess the mitochondrial condition in this study, we employed the membrane‐permeant JC‐1 dye, which exhibits different fluorescent properties depending on the mitochondrial membrane potential.

**FIGURE 8:**
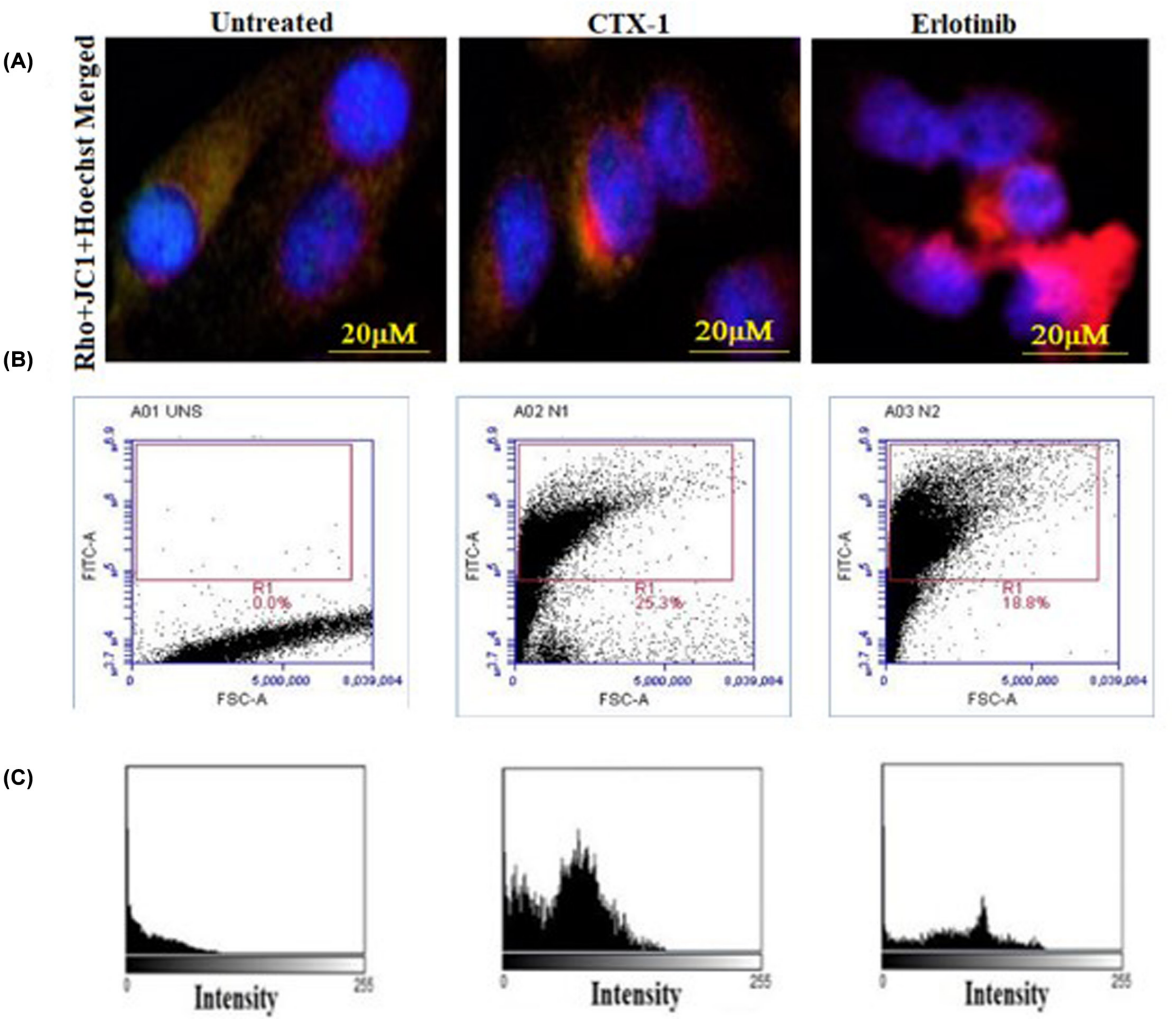
Disruption of Mitochondrial Transmembrane Potential by CTX‐1 and Erlotinib at IC_50_ Concentration in A549 Cell Lines: (A) Rhodamine‐123 and JC‐1 staining were used to assess the mitochondrial transmembrane potential in untreated and treated cells with CTX‐1 and Erlotinib at their IC_50_ concentrations; (B) The percentage of cells exhibiting disrupted mitochondrial transmembrane potential (∆Ψm) was quantified (The graph represents the percentage of cells with disrupted ∆Ψm in the treated groups); (C) The intensity of the mitochondrial transmembrane potential by CTX‐1 and erlotinib at IC_50_ concentration in A549 cell lines and compared with the untreated cells, intensity was found to be higher in CTX‐1 treated A549 cells.

The JC‐1 dye exists in a monomeric form and emits green fluorescence at 529 nm. However, under conditions of high mitochondrial membrane potential, it forms red fluorescent J‐aggregates that accumulate within the mitochondria, leading to a shift in fluorescence emission to 590 nm (Figure 8A). The ratio of fluorescence intensities serves as an indicator of the imbalance in the mitochondrial membrane potential. Using fluorescence microscopy and flow cytometry analysis, we observed a disrupted membrane potential of the mitochondria (Figure 8B,C).

Flow cytometry analysis was performed on JC‐1‐stained cells, enabling visualization and quantification of the disrupted mitochondrial membrane potential. These results indicate that treatment with CTX‐1 and erlotinib at their respective IC_50_ concentrations disrupts mitochondrial transmembrane potential in A549 cell lines. The fluorescence and flow cytometry analyses provided valuable insights into the impact of these compounds on mitochondrial function, further highlighting their potential as therapeutic agents for lung cancer treatment. These findings highlight the impact of the tested compounds on mitochondrial function, providing insights into their potential involvement in the induction of programmed cell death in lung cancer cells. The assessment of mitochondrial membrane potential through JC‐1 staining allows us to gain a deeper understanding of the dynamic interactions between the compounds and mitochondria, shedding light on the mechanisms underlying their anticancer effects (Raw R9 in Data S1).

### 3.9 Induction of apoptosis and enhancement of ROS levels by CTX‐1 and erlotinib in A549 lung cancer cells

**FIGURE 9:**
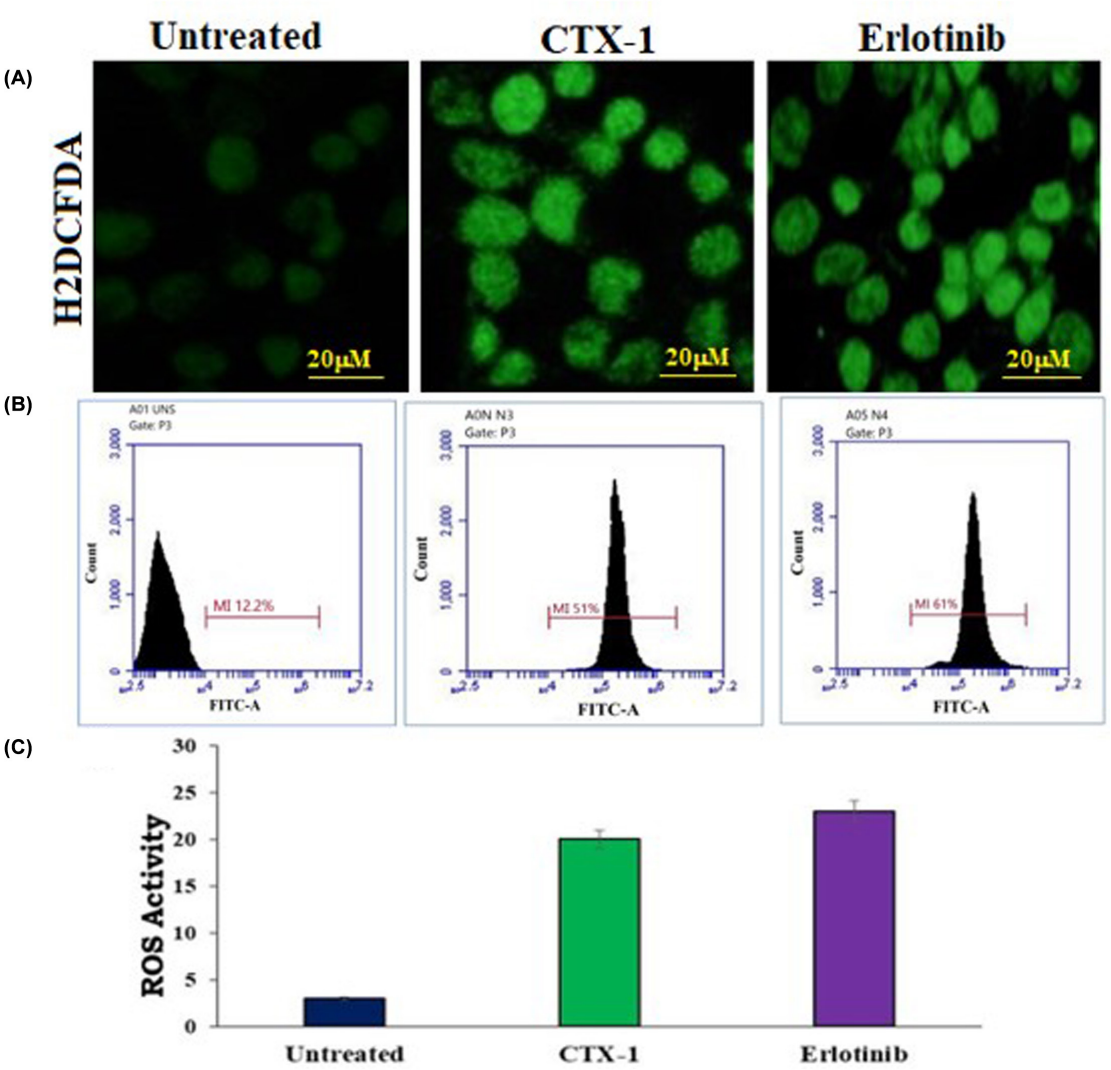
(A) The ROS activity of CTX‐1 and erlotinib were found to be increased compared with the untreated well these results the ROS mediated apoptosis; (B) The flow cytometry was used to determine the percentage of ROS induction was found to be 12.2% in untreated, 51% and 61% showed by CTX‐1 and erlotinib; (C) Next, the ROS fluorescence intensity of untreated, treated by CTX‐1 and erlotinib against A549 cell line were represented.

To gain deeper insights into the underlying mechanisms of apoptosis induction in cancer cells, we examined the impact of CTX‐1 and erlotinib on the generation of reactive oxygen species (ROS). ROS levels were quantified using the 2′,7′‐dichlorofluorescin diacetate (DCFDA) probe, which allows for the detection of intracellular ROS. After treating A549 lung cancer cells with CTX‐1 and erlotinib for 48 h, we observed a significant increase in the intensity of green fluorescence compared with the untreated cells (Figure 9). This pronounced fluorescence enhancement indicated a substantial elevation in ROS levels induced by both CTX‐1 and erlotinib treatments, strongly suggesting the involvement of ROS in the induction of apoptosis. To further validate the association between increased ROS levels and induction of apoptosis, we employed a positive control by treating A549 cells with H_2_O_2_ (5 μM), a known inducer of ROS generation. As anticipated, these cells exhibited substantially higher levels of ROS, as indicated by the more intense green fluorescence signal. This correlation between elevated ROS levels and the induction of apoptosis further strengthens the significance of ROS‐mediated pathways in the cytotoxic effects of CTX‐1 and erlotinib (Raw R10 in Data S1).

These findings provide substantial evidence for the involvement of ROS in the apoptotic pathways induced by CTX‐1 and erlotinib in A549 lung cancer cells. The significant increase in ROS levels upon treatment with these agents suggests their potential as therapeutic candidates that exploit ROS‐mediated mechanisms to selectively induce cancer cell death. Further investigations into the precise molecular interactions and downstream signalling pathways associated with ROS generation will enhance our understanding of the anticancer effects of CTX‐1 and erlotinib and may open new avenues for targeted lung cancer therapies.

### 3.10 Inhibition of colony formation by CTX‐1 and erlotinib in A549 lung cancer cells: demonstration of anticancer activity and determination of CTX‐1's IC_50_ Value

**FIGURE 10:**
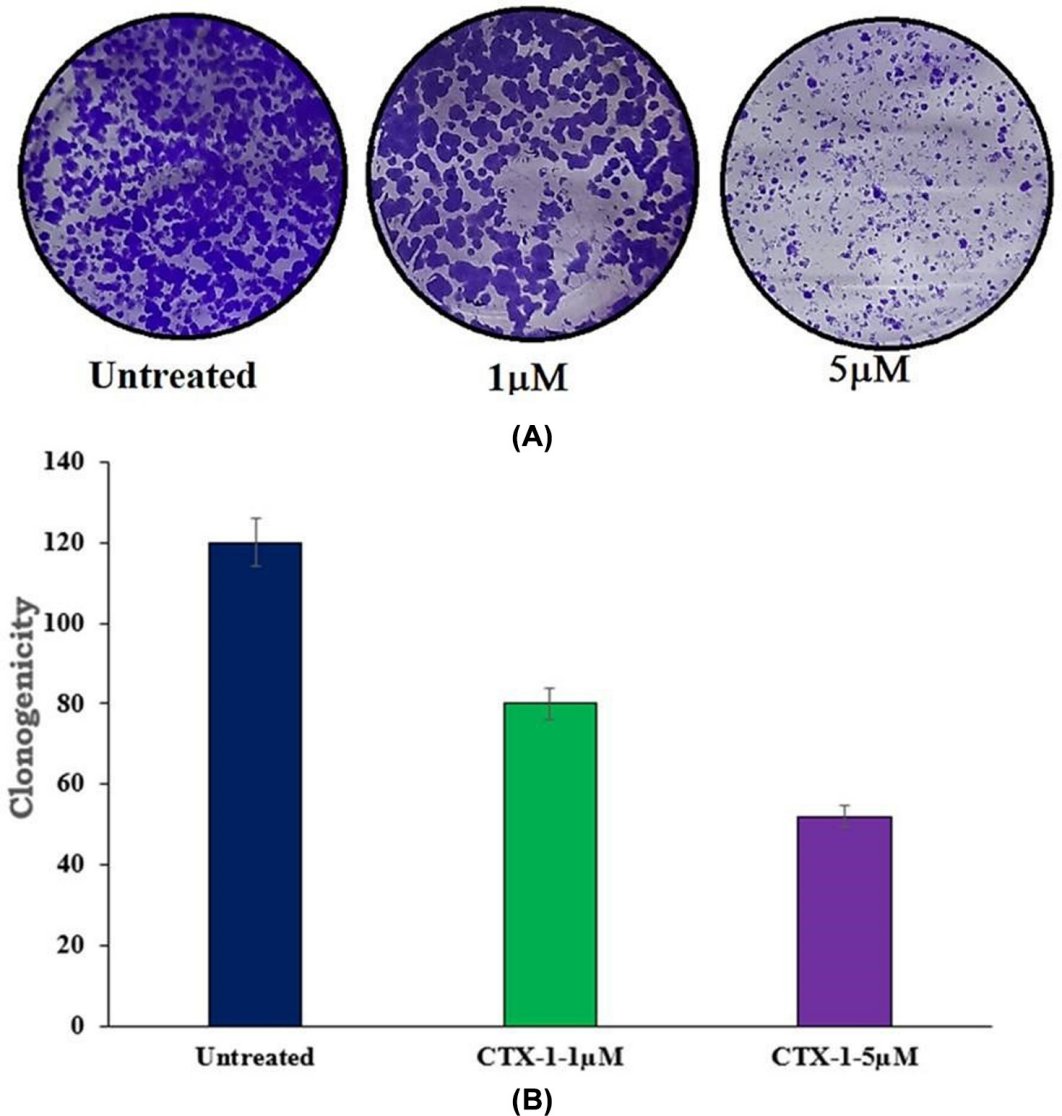
(A) Treatment with the CTX‐1 inhibits colony development. The chemical was applied in increasing concentrations (1 μM, 5 μM) to the triple‐negative cancer cell line A549 (When compared to cells that were not treated, the chemical significantly reduced the number of colonies that formed). (B) A drastic decrease in colony formation was observed after treatment with CTX‐1 and erlotinib and the intensity is represented.

The inhibitory effects of CTX‐1 and erlotinib on colony formation in the lung cancer cell line A549 were investigated to assess their anticancer activity. Increasing concentrations of the drugs (1–10 μM) were administered to the cells for 10 days to facilitate colony growth. Quantification of colonies was performed using Image‐J software. The results demonstrated concentration‐dependent inhibition of colony formation. The treated cells exhibited a significantly reduced number of colonies compared with the untreated cells, indicating the potent inhibitory effect of CTX‐1 and erlotinib on colony development. Notably, CTX‐1 exhibited remarkable cytotoxicity activity, as evidenced by an IC_50_ value of 1.5 mM determined from the results of the clonogenic experiment (Figure 10; Raw R11 in Data S1).

These findings highlight the ability of CTX‐1 and erlotinib to suppress colony formation in lung cancer cells, providing evidence of their potential as effective therapeutic agents. The concentration‐dependent inhibition of colony development further emphasises their dose‐dependent cytotoxic effects. The determination of the IC_50_ value for CTX‐1 underscores its potency in inhibiting colony formation and supports its potential as a promising candidate for further investigation in lung cancer treatment.

### 3.11 Expression of EGFR on CTX‐1‐treated lung cancer cells

**FIGURE 11:**
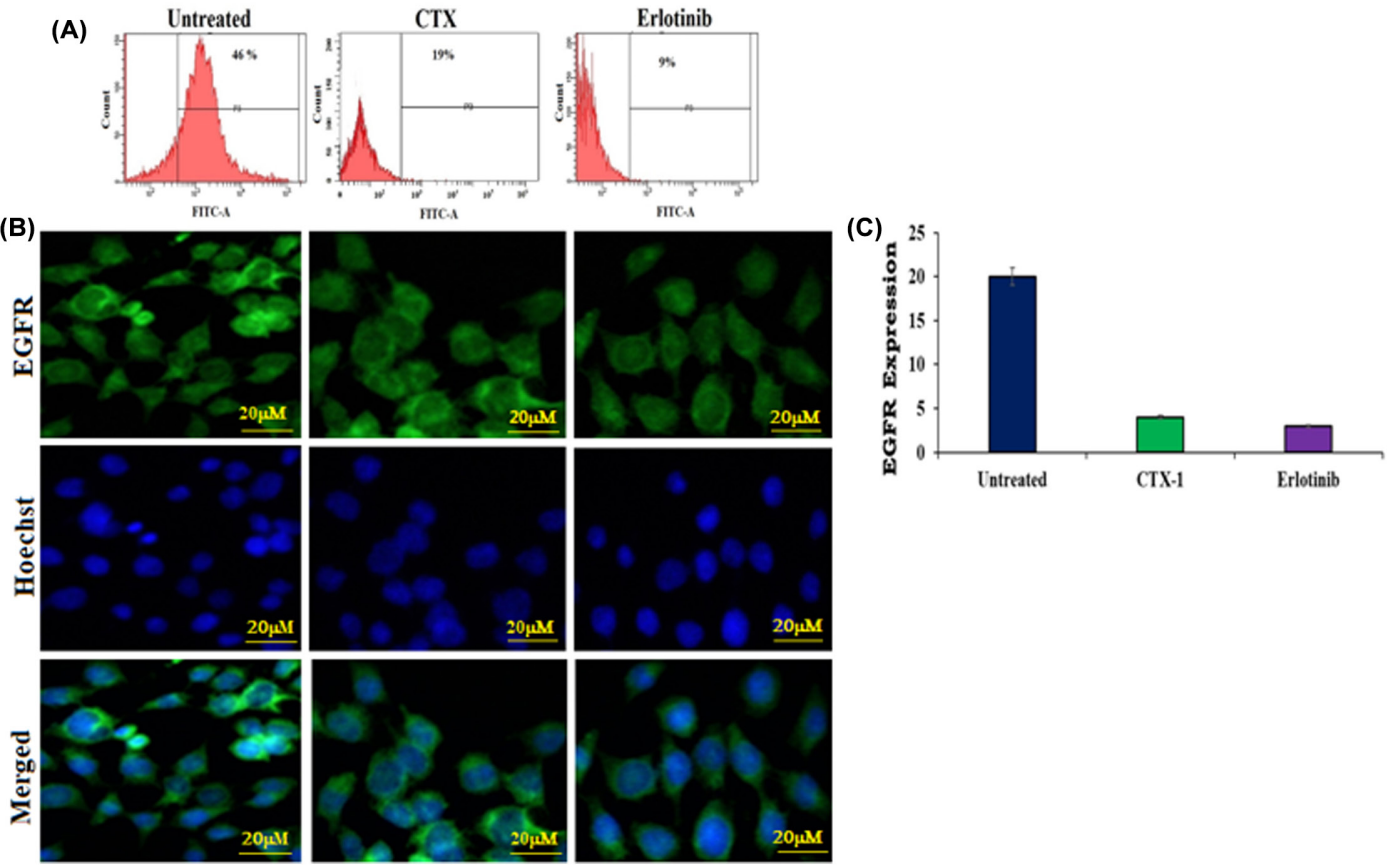
EGFR expression and internalization in CTX‐1‐ and erlotinib‐treated A549 cells: (A) Immunofluorescence microscopy analysis depicting the internalisation of CPPFITC into EGFR‐expressing A549 cells. (B) The untreated cells exhibited significantly higher EGFR expression levels, whereas cells treated with CTX‐1 and erlotinib demonstrated minimal fluorescence signal, indicating reduced EGFR expression (magnification 200×, scale bar 20 μm); (C) Quantification of EGFR expression in the untreated and treated groups, revealing the impact of CTX‐1 and erlotinib treatment on EGFR expression levels.

To evaluate the impact of CTX‐1 treatment on EGFR expression, we examined EGFR levels in lung cancer cells. Immunofluorescence analysis was performed using a FITC‐labelled anti‐EGFR monoclonal antibody (MoAb). Both untreated and CTX‐1‐treated A549 cells were observed by fluorescence microscopy. The results revealed a significant disparity in EGFR expression between the untreated and treated cells. Notably, CTX‐1‐treated cells exhibited higher fluorescence intensity for EGFR expression compared with untreated cells (Figure 11A,B). These findings indicate that CTX‐1 treatment increases EGFR expression in lung cancer cells, potentially suggesting alterations in EGFR signalling pathways. The observed upregulation of EGFR expression provides valuable insights into the molecular effects of CTX‐1 on lung cancer cells, which may involve modifications in cellular signalling and a potential augmentation of sensitivity to the anticancer effects of CTX‐1 (Raw R12 in Data S1).

These findings provide insights into the internalisation process of CPPFITC into EGFR‐expressing A549 cells and highlight the influence of CTX‐1 and erlotinib treatment on EGFR expression. The reduced fluorescence signal in treated cells suggests the potential internalisation and altered distribution of EGFR because of the treatment. These observations contribute to our understanding of the cellular response to CTX‐1 and erlotinib and their effects on EGFR expression dynamics.

These results contribute to our understanding of the mechanism of action of CTX‐1 and its potential as a targeted therapy for lung cancer. Further investigations are warranted to explore the functional implications of increased EGFR expression and its role in mediating the response to CTX‐1 treatment. Understanding the relationship between CTX‐1, EGFR expression, and downstream signalling pathways will enable the development of more effective and personalised treatment strategies for patients with lung cancer.

## 4 DISCUSSION

The data gathered in this study indicate an enhanced anticancer capacity of CTX‐1 in comparison with erlotinib, the standard drug for lung cancer treatment. This is manifested through various outcomes, including a decrease in cancer cell viability, initiation of DNA damage, cell cycle suspension, production of ROS, perturbation of mitochondrial transmembrane potential and obstruction of EGFR‐TKD. These observations emphasise CTX‐1's potential as a powerful therapeutic tool in the management of lung cancer. This research uncovers promising evidence regarding the therapeutic capabilities of DAC and its derivative CTX‐1 in the context of lung cancer. CTX‐1's pronounced effect on reducing lung cancer cell viability points to its proficiency in curbing tumour proliferation. The initiation of DNA damage indicates that CTX‐1 may induce DNA fragmentation, thereby hampering the replication and survival mechanisms of cancer cells. The observed cell cycle suspension further endorses the antiproliferative effects of CTX‐1, suggesting its capability to obstruct unrestrained division and growth of cancer cells. The generation of ROS under CTX‐1 treatment underscores its potential to induce oxidative stress in cancer cells, thereby potentially triggering apoptosis via ROS‐mediated pathways.

In addition, CTX‐1's influence on the mitochondrial transmembrane potential demonstrates its impact on mitochondrial function, a factor closely linked with apoptosis. The inhibition of EGFR‐TKD hints at its potential to target critical signalling pathways implicated in lung cancer progression. Comparisons with erlotinib highlight the superior efficacy of CTX‐1. Erlotinib's higher binding affinity compared with CTX‐1 suggests opportunities for optimising CTX‐1's binding interactions with the target protein. The comparative stability between CTX‐1 and erlotinib, established through stability analysis and molecular dynamics simulations, reaffirms CTX‐1's potential as a stable and effective therapeutic agent.

The novelty of this investigation lies in the exploration of DAC and CTX‐1 as potential treatment options for lung cancer, addressing an area that has previously received limited attention despite encouraging early results. A detailed assessment of CTX‐1's biological impacts, including its effects on cell viability, DNA damage induction, cell cycle suspension, ROS production, mitochondrial dysfunction and EGFR‐TKD inhibition, provides a comprehensive understanding of its anticancer activity. These results contribute to the development of novel and potent anticancer treatments, offering promising prospects for lung cancer treatment.

In summary, this study's outcomes underscore the potency of CTX‐1 as an anticancer agent for lung cancer treatment. The superior anticancer activity of CTX‐1 compared with that of erlotinib, alongside its impact on critical cellular processes and signalling pathways, supports its potential as a promising therapeutic candidate. Future investigations and optimization of CTX‐1 hold promise for propelling the field of lung cancer treatment and enhancing patient outcomes.

## 5 CONCLUSION

This study presents compelling insights into the potential of 3,6‐diaminoacridine‐9‐carbonitrile (DAC) and its derivative CTX‐1 as potential avenues for lung cancer therapy. Through a detailed analysis of key factors such as binding affinity, stability and biological effects, we have highlighted the ability of CTX‐1 to impede tumour proliferation and instigate apoptosis in lung cancer cell lines. Our findings indicate that CTX‐1 exhibits superior anticancer properties compared with the commonly used drug erlotinib, as indicated by its significant impact on decreasing cancer cell viability, instigating DNA damage, halting cell cycle progression, enhancing reactive oxygen species (ROS) production, altering mitochondrial transmembrane potential and inhibiting EGFR‐TKD.

The novelty of this research lies in its unique focus areas. While the effectiveness of DAC against glioblastoma is widely recognised, our investigation paves the way for its application in lung cancer therapy—a field that, despite promising initial results, has been relatively underexplored. In addition, our in‐depth examination of CTX‐1, a derivative of DAC, and its comparison with erlotinib in terms of binding affinity and stability provides new insights into CTX‐1's potential as a lung cancer therapeutic. Our comprehensive evaluation of CTX‐1's biological impacts on lung cancer cell lines using mechanistic approaches contributes to a more profound understanding of its mechanisms of action and reaffirms its potential as a potent anticancer agent.

This study emphasizes the pivotal roles of mitochondrial dysfunction and ROS generation in the apoptotic pathways triggered by CTX‐1 and erlotinib. Our study of their effects on mitochondrial transmembrane potential and ROS levels extends our understanding of the molecular mechanisms underlying lung cancer cell death. Collectively, the insights gained from this research significantly advance the field of novel and powerful anticancer agents and hold promise for future lung cancer treatment strategies. Comprehensive analysis of CTX‐1—including its binding affinity, stability and biological effects, coupled with its superior anticancer activity compared with erlotinib—highlights it as a promising candidate for further investigation in preclinical and clinical stages. This study sets a solid foundation for future research initiatives aimed at improving lung cancer therapies and patient outcomes.

### 5.1 Limitations of the study

Despite the valuable insights provided by this study, it is essential to acknowledge its limitations to ensure a comprehensive understanding of the findings. The research methodology involved in vitro tests on lung cancer cell lines, a system that may not completely mirror the intricate tumour microenvironment and dynamic interactions with adjacent tissues. The next logical progression in this research would be the integration of animal models or clinical trials to authenticate the effectiveness and safety of CTX‐1 in a setting that closely mirrors the human body. While our data show that CTX‐1 outperforms erlotinib in terms of anticancer activity, we acknowledge the need for further refinement of CTX‐1's binding affinity and stability to maximise its therapeutic potential. This enhancement process may necessitate structural adjustments or formulation tactics to improve its bioavailability and specificity towards the target.

This investigation focuses on the biological effects of CTX‐1. Therefore, comprehensive assessments of its pharmacokinetics, toxicological profile and potential interactions with other drugs are crucial for its translation into clinical practice. A thorough knowledge of CTX‐1's pharmacological attributes and possible adverse effects is crucial to guaranteeing its safe and effective use in patients.

Despite the stated limitations, the findings of this investigation provide a meaningful basis for considering CTX‐1 as a potential therapeutic approach for lung cancer. By addressing the outlined limitations in upcoming research projects, we can deepen our understanding of CTX‐1 and facilitate its development as a unique and effective therapeutic strategy for lung cancer patients.

## AUTHOR CONTRIBUTIONS

**Rajesh Kumar Meher:** Conceptualization (equal); investigation (equal); methodology (equal); writing – original draft (equal). **Showkat Ahmad Mir:** Conceptualization (equal); funding acquisition (equal); supervision (equal); writing – review and editing (equal). **Kritika Singh:** Project administration (equal); resources (equal); validation (equal); writing – original draft (equal). **Nobendu Mukerjee:** Formal analysis (equal); investigation (equal); methodology (equal); validation (equal); writing – original draft (equal). **Binata Nayak:** Project administration (equal); resources (equal); validation (equal); writing – original draft (equal). **Ajoy Kumer:** Conceptualization (equal); resources (equal); supervision (equal); writing – review and editing (equal). **Torki A. Zughaibi:** Investigation (equal); resources (equal); writing – original draft (equal). **Mohd Shahnawaz Khan:** Formal analysis (supporting); resources (supporting); writing – review and editing (supporting). **Shams Tabrez:** Funding acquisition (equal); project administration (supporting); supervision (equal); validation (supporting); writing – review and editing (equal).

## CONFLICT OF INTEREST STATEMENT

All authors declare that no conflict of interest exists.

## CONSENT TO PUBLISH

All authors consented to the publication of this work. The authors all confirm the permission for publication for this research work.

## Supporting information


Appendix S1.

Data S1.

## Data Availability

We declare that all the data generated are included in this study.
