# Supplementary material for: Decoding dynamic interactions between EGFR‐TKD and DAC through computational and experimental approaches: A novel breakthrough in lung melanoma treatment
Source: J Cell Mol Med. 2024 Apr 29;28(9):e18263. doi: 10.1111/jcmm.18263 (PMC11058330; doi:10.1111/jcmm.18263)
Supplement: Supplementary file 1 — Appendix S1. [file JCMM-28-e18263-s002.docx]

**Decoding dynamic interactions between EGFR-TKD & DAC through computational and experimental approaches: Novel breakthrough in lung melanoma treatment**

**Supplementary File**

Rajesh Kumar Meher^1†^, **Showkat Ahmad Mir**^1†*^, Kritika Singh^2†^, Nobendu Mukerjee^3,4†^, Binata Nayak^1^, **Ajoy Kumer^5*^**, Torki A. Zughaibi^6,7^, Mohd Shahnawaz Khan^8^, **Shams Tabrez^6,7*^**

^1^School of Life Sciences, Sambalpur University, Burla-768019, Odisha, India.

(RKM: [rajeshkumarmeher99@suniv.ac.in](mailto:rajeshkumarmeher99@suniv.ac.in)), (SAM: [showkat@suniv.ac.in](mailto:showkat@suniv.ac.in)), (BN: [binatanayak@suniv.ac.in](mailto:binatanayak@suniv.ac.in))

^2^Offenburg University of Applied Sciences, Offenburg, Germany (KS: [kritikaamity29@gmail.com](mailto:kritikaamity29@gmail.com))

^3^Center for Global Health Research, Saveetha Medical College and Hospital, Saveetha Institute of Medical and Technical Sciences, Chennai, India. (NM: [nabendu21@rkmvccrahara.org](mailto:nabendu21@rkmvccrahara.org))

^4^Department of Health Sciences, Novel Global Community Educational Foundation, Hebersham, NSW, Australia. (NM: [nabendu21@rkmvccrahara.org](mailto:nabendu21@rkmvccrahara.org))

^5^Department of Chemistry, College of Arts and Sciences IUBAT-International University of Business Agriculture and Technology, Dhaka 1230, Bangladesh. (AK: [kumarajoy.cu@gmail.com](mailto:kumarajoy.cu@gmail.com))

^6^King Fahd Medical Research Center, King Abdulaziz University, Jeddah, Saudi Arabia

^7^Department of Medical Laboratory Sciences, Faculty of Applied Medical Sciences, King Abdulaziz University, Jeddah, Saudi Arabia; (MST: [shamstabrez1@gmail.com](mailto:shamstabrez1@gmail.com); TAZ: [zughaibitorki@gmail.com](mailto:zughaibitorki@gmail.com))

^8^Department of Biochemistry, College of Science, King Saud University, Riyadh, Saudi Arabia; (MSK: [moskhan@ksu.edu.sa](mailto:moskhan@ksu.edu.sa))


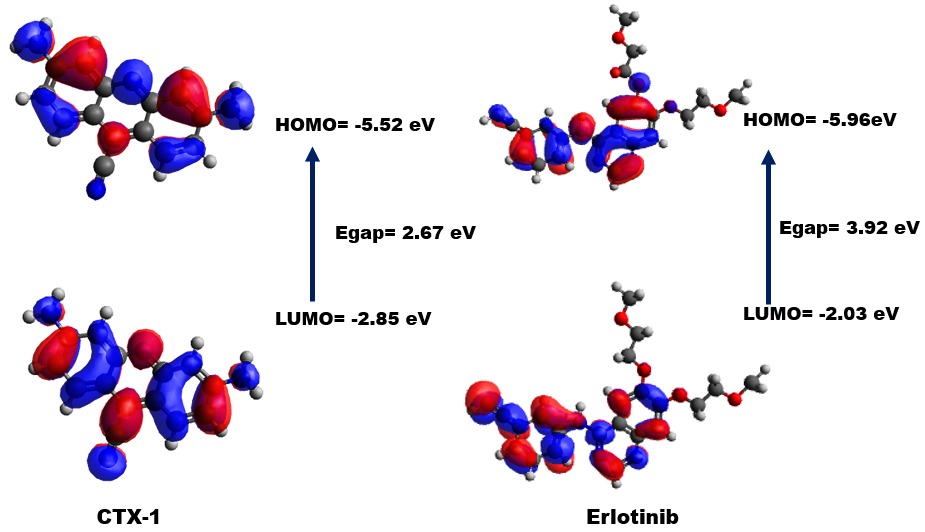


**Figure S1:** The HOMO and LUMO of CTX-1 and erlotinib were determined using the DFT method.

**Table S1:** Quantum calculations of CTX-1 and erlotinib were determined by the ORCA program.

| **Ligand** | **E_HOMO_ (eV)** | **E_LUMO_ (eV)** | **E_gap_ (eV)** | **I = -E_HOMO_ (eV)** | **A = -E_LUMO_ (eV)** | **μ = -I + A/2** | **χ = I + A/2** | **η = I-A/2** | **σ = 1/η** | **ω = μ2/2η** |
| --- | --- | --- | --- | --- | --- | --- | --- | --- | --- | --- |
| AQ44 | -5.96 | -2.03 | 3.92 | 5.96 | 2.03 | -3.99 | 3.99 | 1.96 | 0.50 | 4.07 |
| CTX-1 | -5.52 | -2.85 | 2.67 | 5.52 | 2.85 | -4.19 | 4.19 | 1.33 | 0.74 | 6.57 |

**
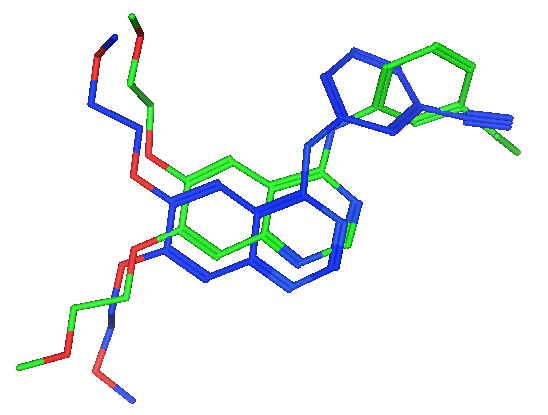
**

**Figure S2. Docking of native ligand with the experimental TKD bound erlotinib. The docked ligand is represented in blue color and the co-crystal ligand is represented in green color.**


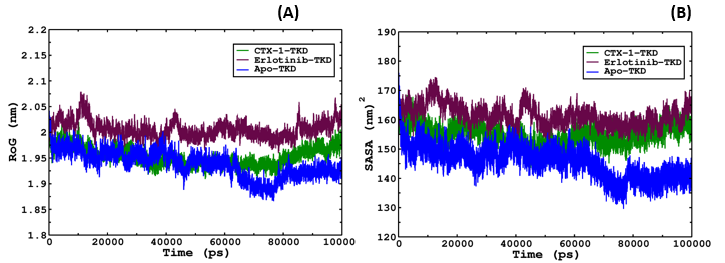


**Figure S3:** The radius of gyrations of TKD bound CTX-1 and the reference molecule is represented in green and maroon, and the trajectory of the free TKD is represented in blue. SASA was calculated to determine the protein folding of both complexes complexed with CTX-1 and erlotinib
