# Supplementary material for: Decoding dynamic interactions between EGFR‐TKD and DAC through computational and experimental approaches: A novel breakthrough in lung melanoma treatment
Source: J Cell Mol Med. 2024 Apr 29;28(9):e18263. doi: 10.1111/jcmm.18263 (PMC11058330; doi:10.1111/jcmm.18263)
Supplement: Supplementary file 2 — Data S1. [file JCMM-28-e18263-s001.zip › R10-ROS.pdf]

Raw Data ROS activity

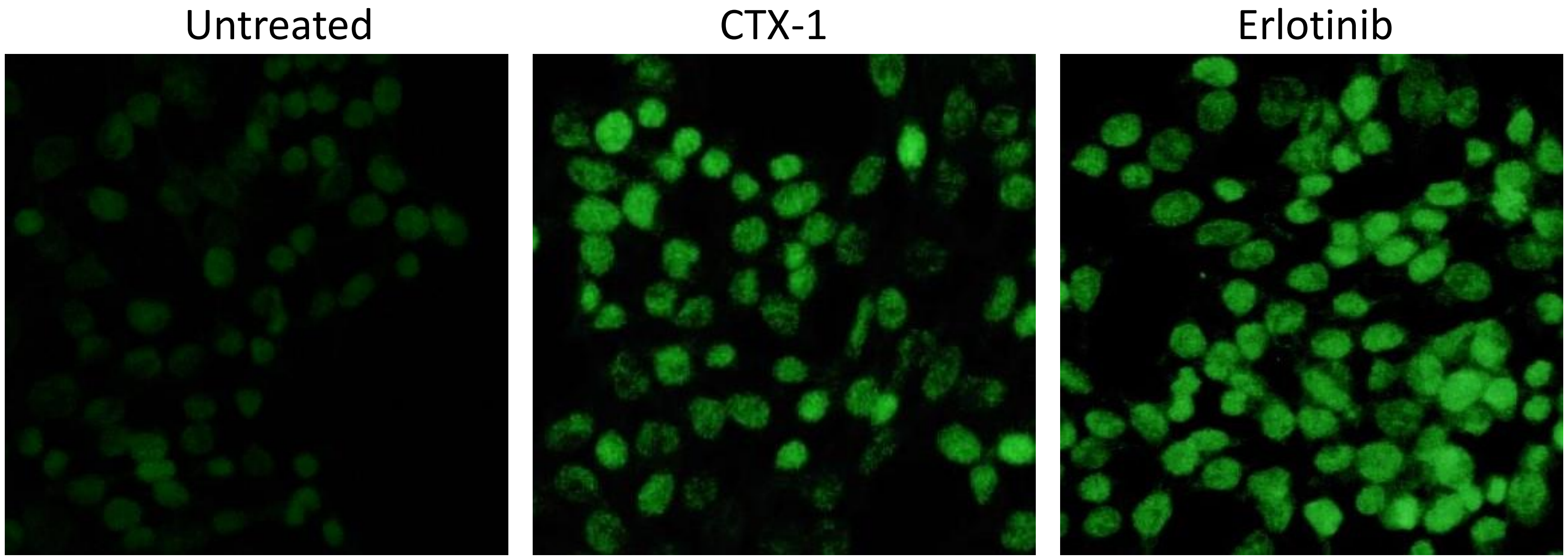

Processed Data for presentation ROS activity

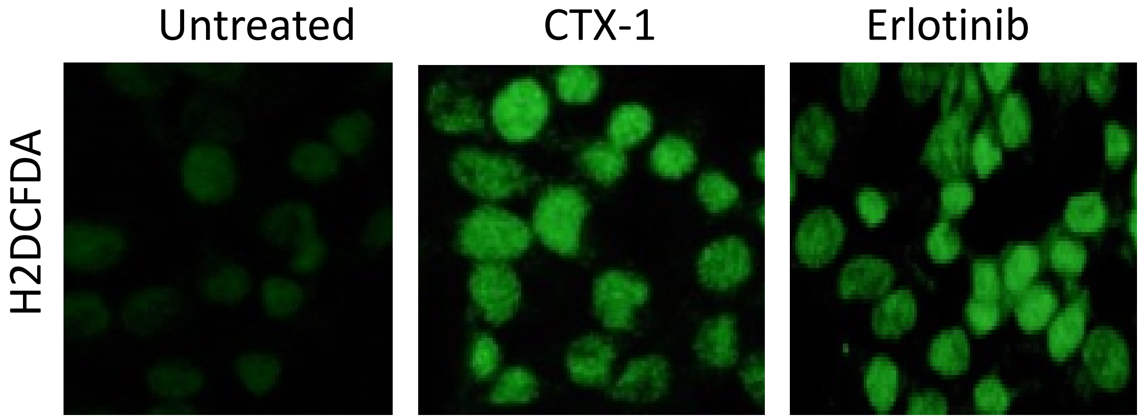

Flow cytometry Raw Data ROS activity

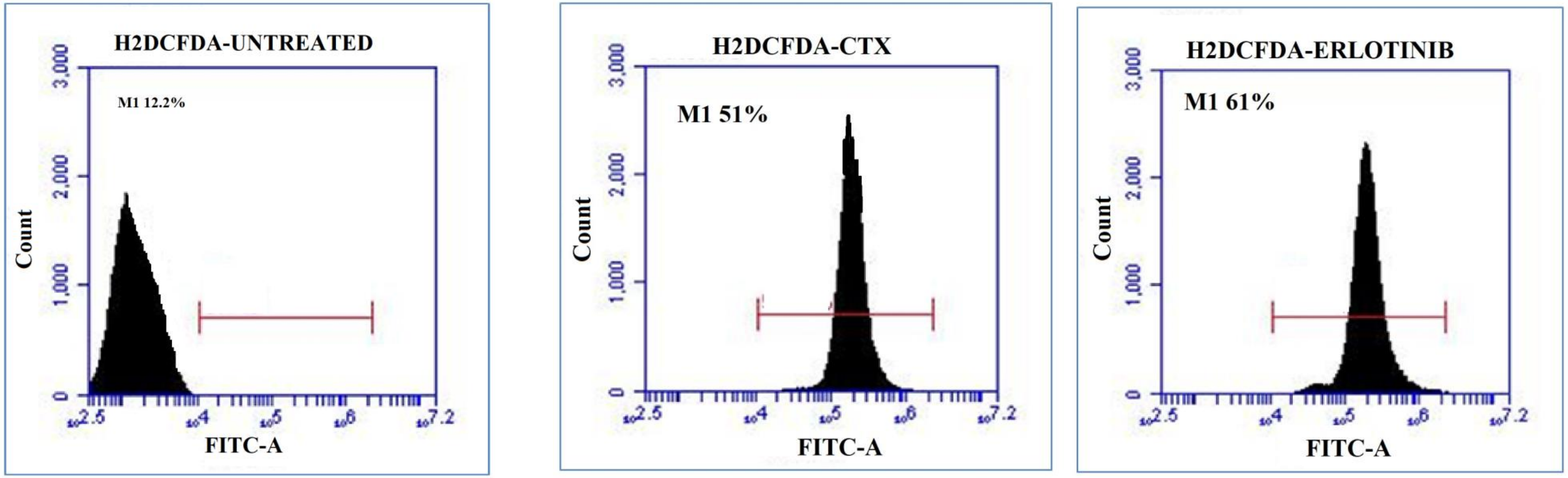

Flow cytometry Processed Data for presentation ROS activity

Untreated **CTX-1** **Erlotinib**

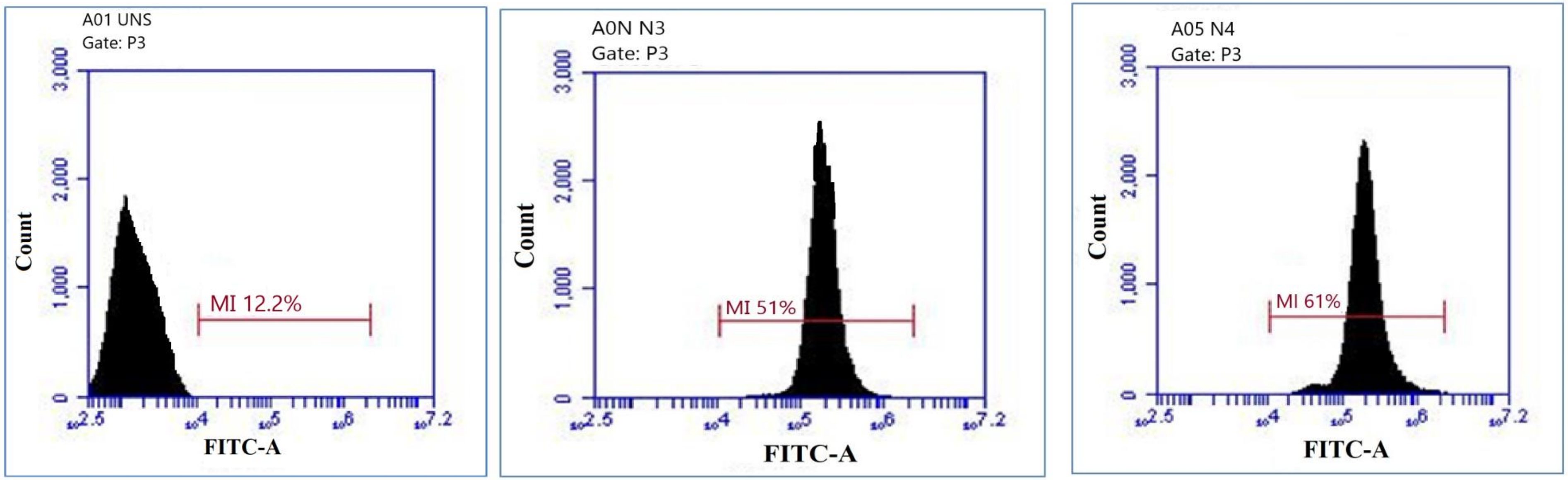

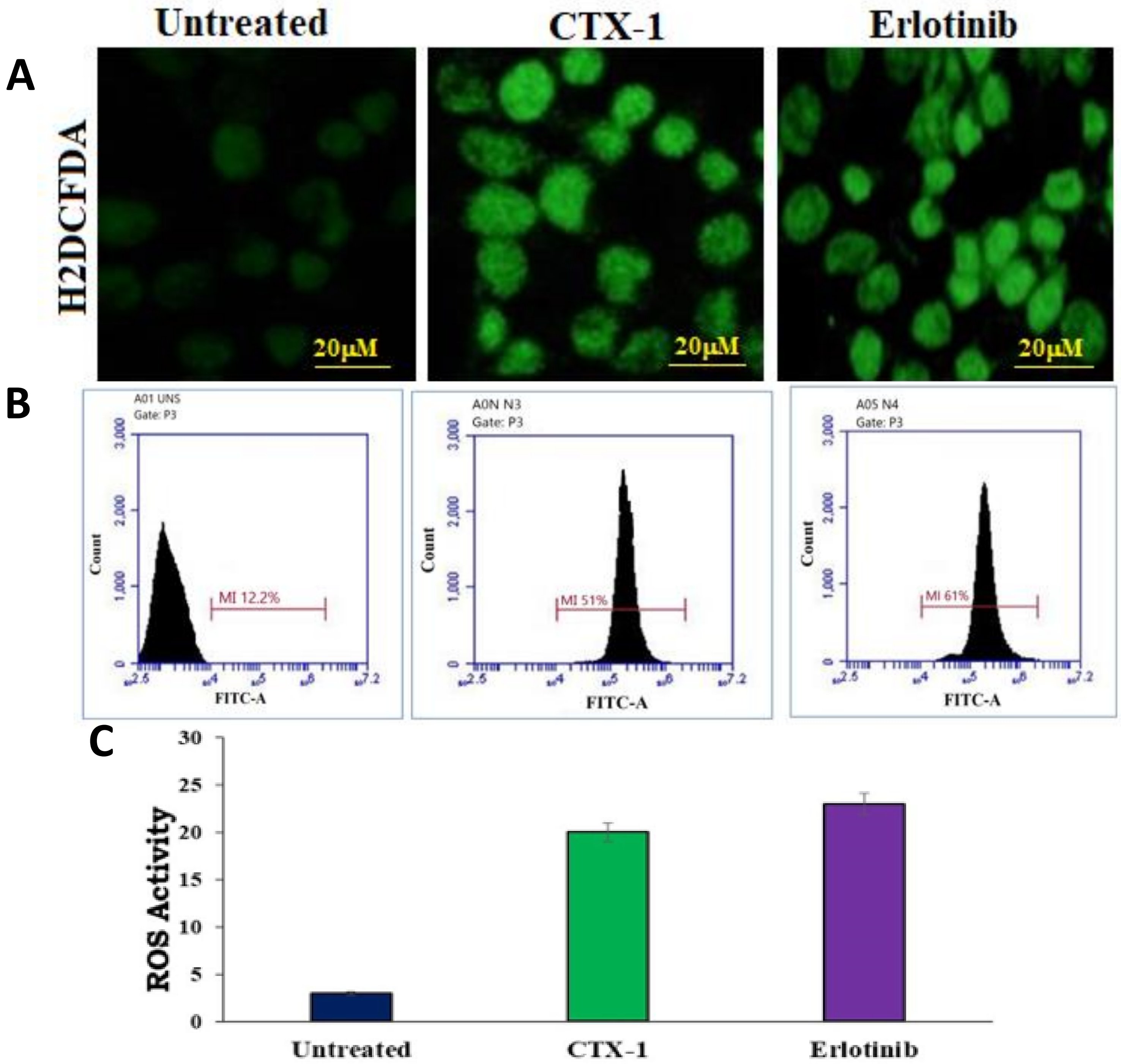

Merged all data for Presentation
