# Supplementary material for: Decoding dynamic interactions between EGFR‐TKD and DAC through computational and experimental approaches: A novel breakthrough in lung melanoma treatment
Source: J Cell Mol Med. 2024 Apr 29;28(9):e18263. doi: 10.1111/jcmm.18263 (PMC11058330; doi:10.1111/jcmm.18263)

Raw Data of EGFR Expression

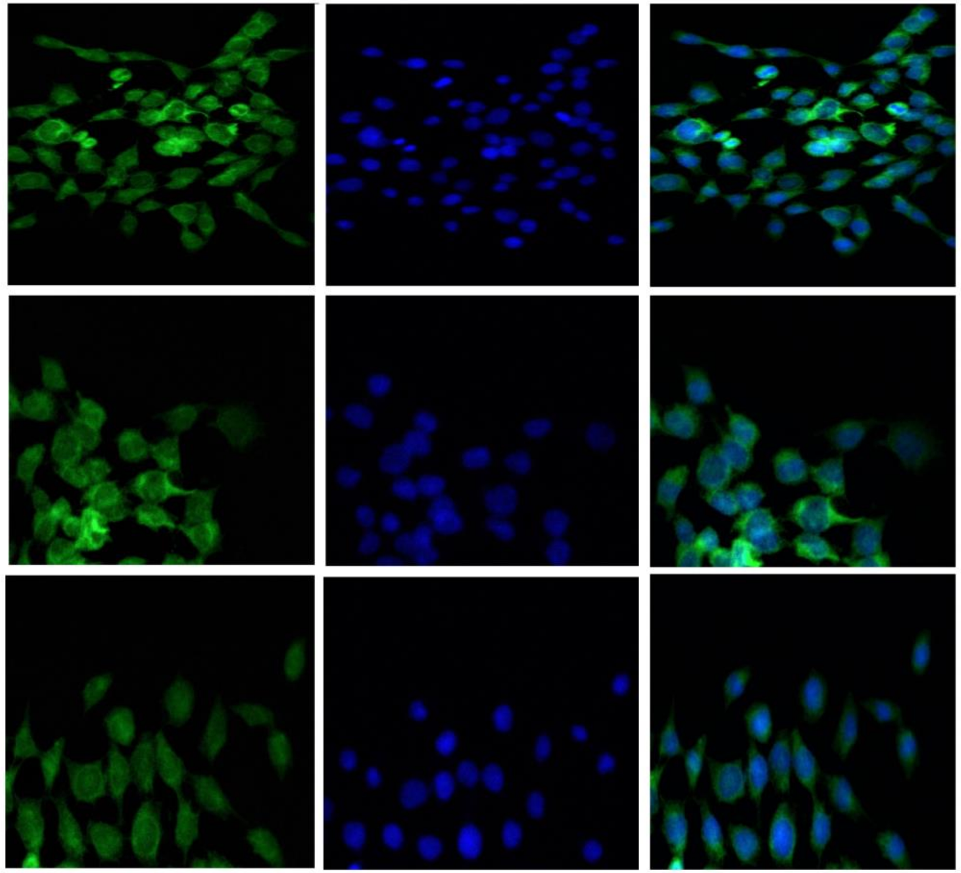

Processed Data of EGFR Expression

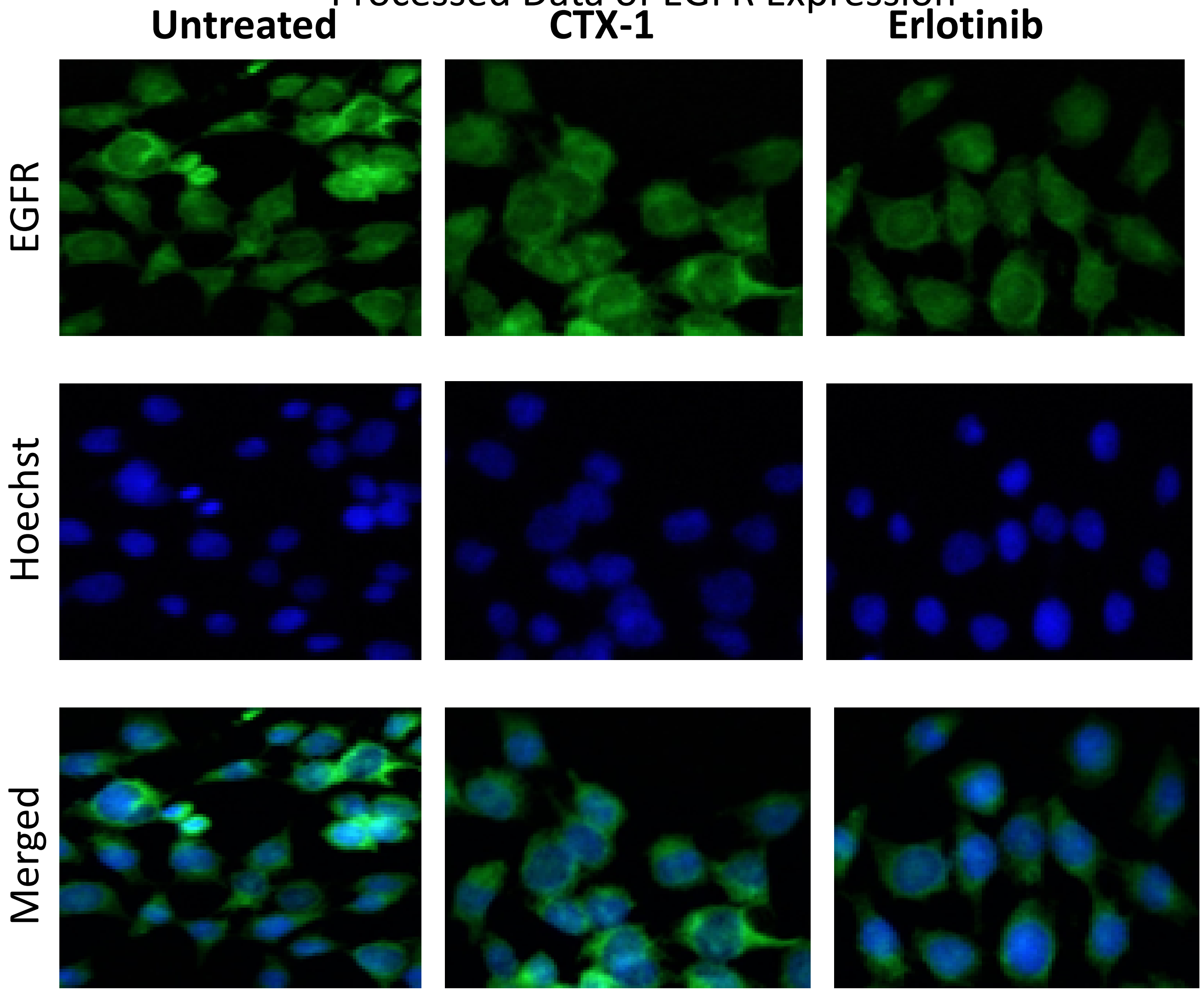

Flow cytometry Raw Data of EGFR Expression

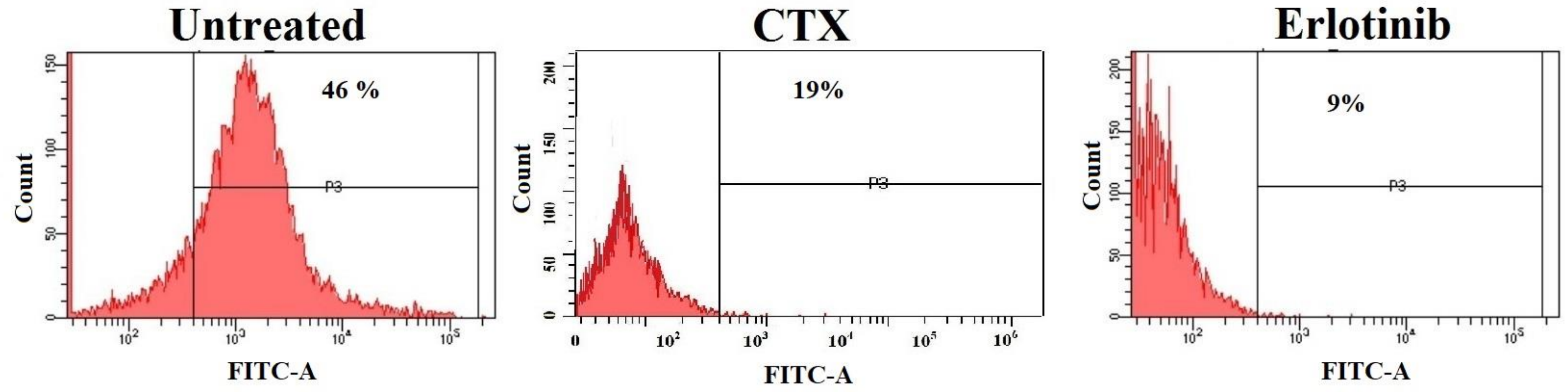

	Untreated	CTX-1	Erlotinib
All events	100	100	100
P3	46	19	9

Processed Flow cytometry Data of EGFR Expression

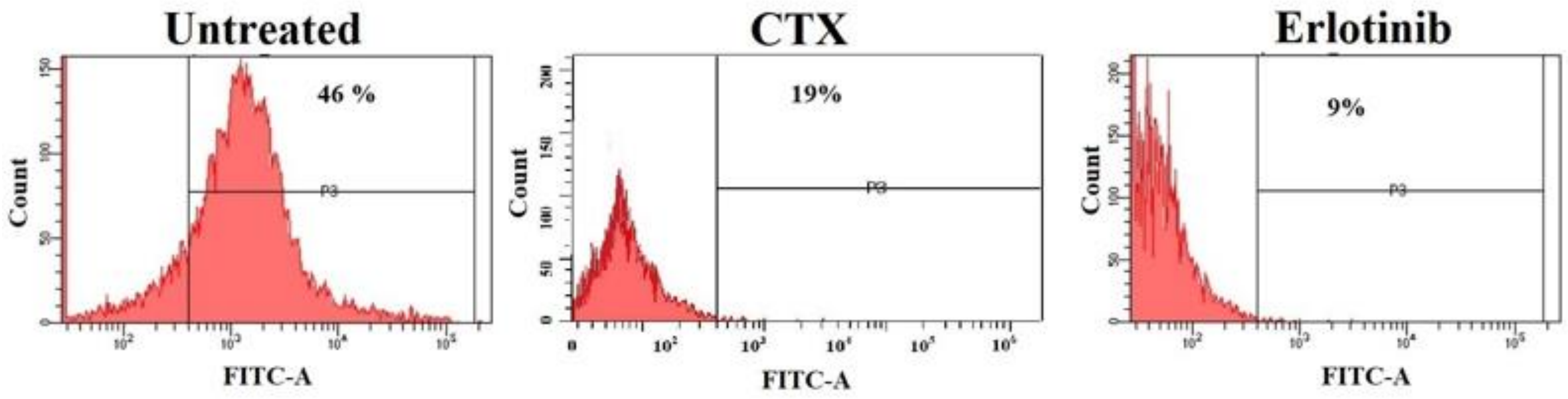

Merged all data for presentation

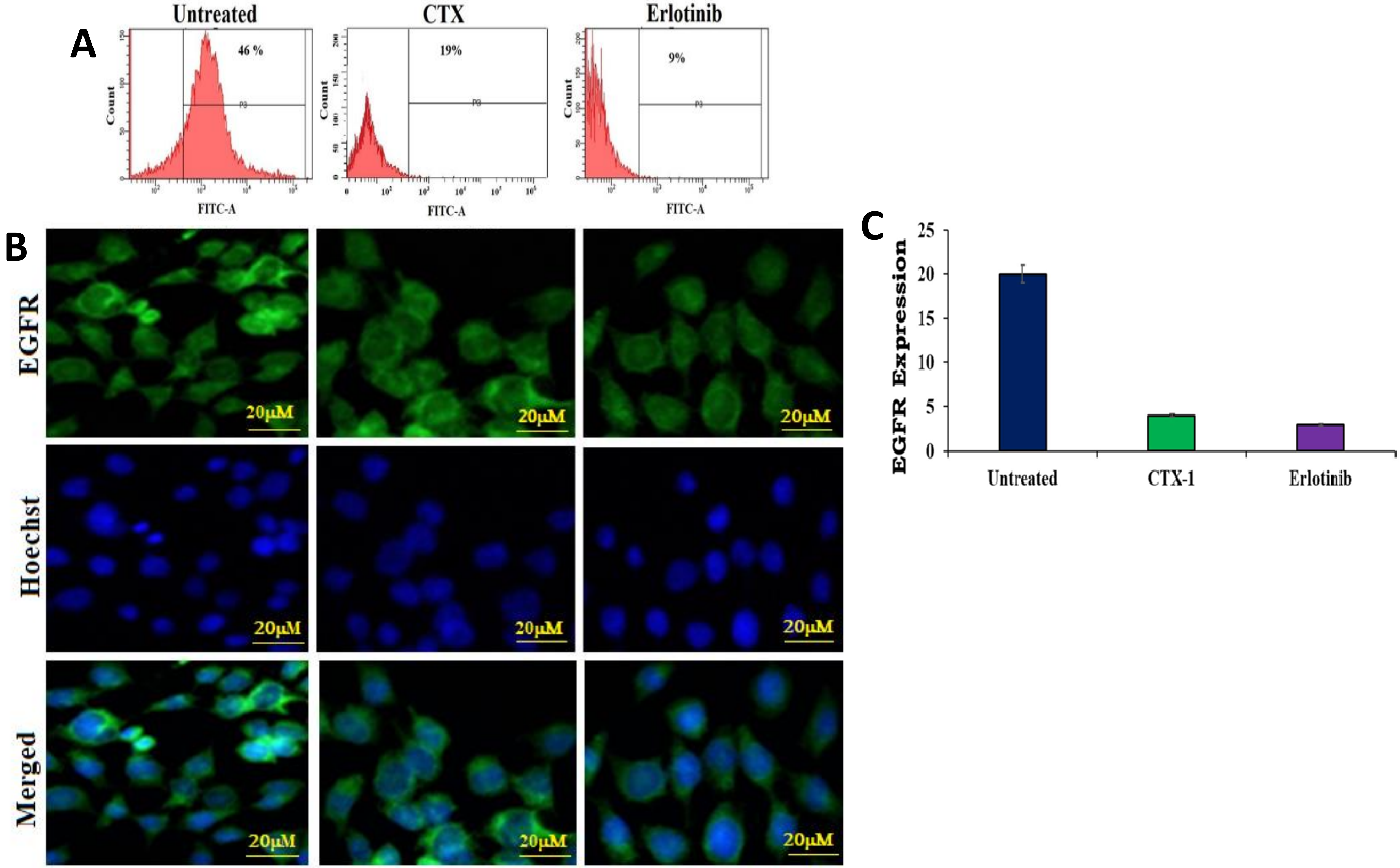

Supplement: Supplementary file 2 — Data S1. [file JCMM-28-e18263-s001.zip › R12-EGFR Expression.pdf]
