# Supplementary material for: Decoding dynamic interactions between EGFR‐TKD and DAC through computational and experimental approaches: A novel breakthrough in lung melanoma treatment
Source: J Cell Mol Med. 2024 Apr 29;28(9):e18263. doi: 10.1111/jcmm.18263 (PMC11058330; doi:10.1111/jcmm.18263)

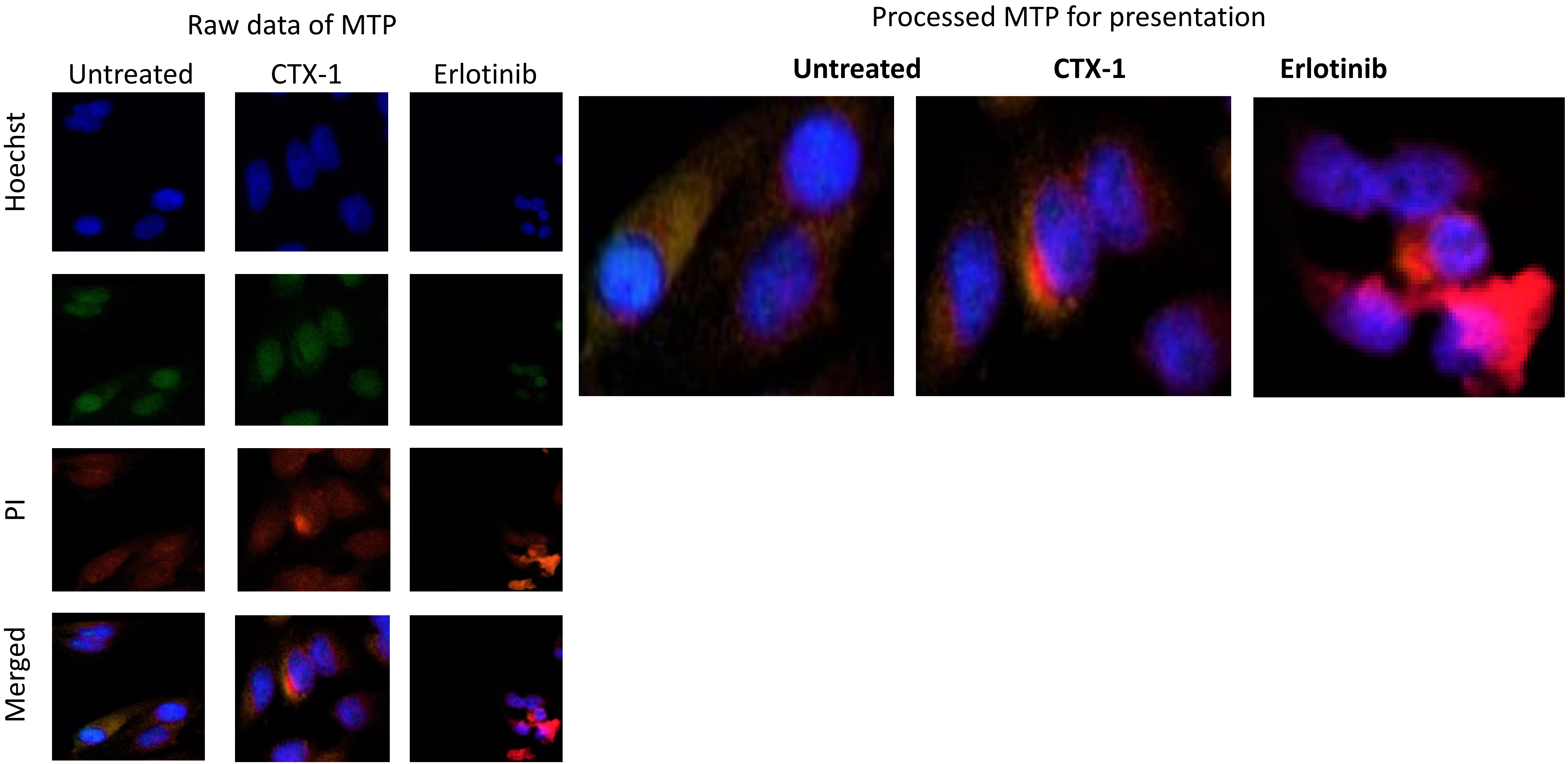
Raw data of MTP
Processed MTP for presentation
Untreated
CTX-1
Erlotinib
Hoechst
PI
Merged
Untreated
CTX-1
Erlotinib

Flow cytometry data Raw data of MTP

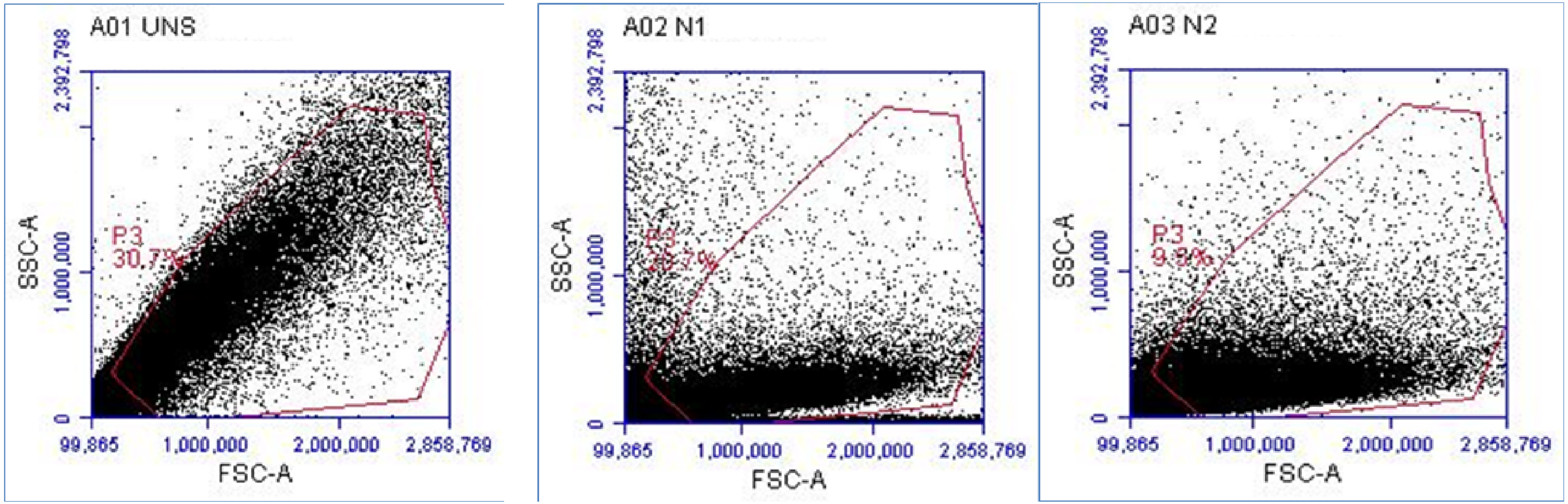

Processed flow cytometry data of MTP for presentation

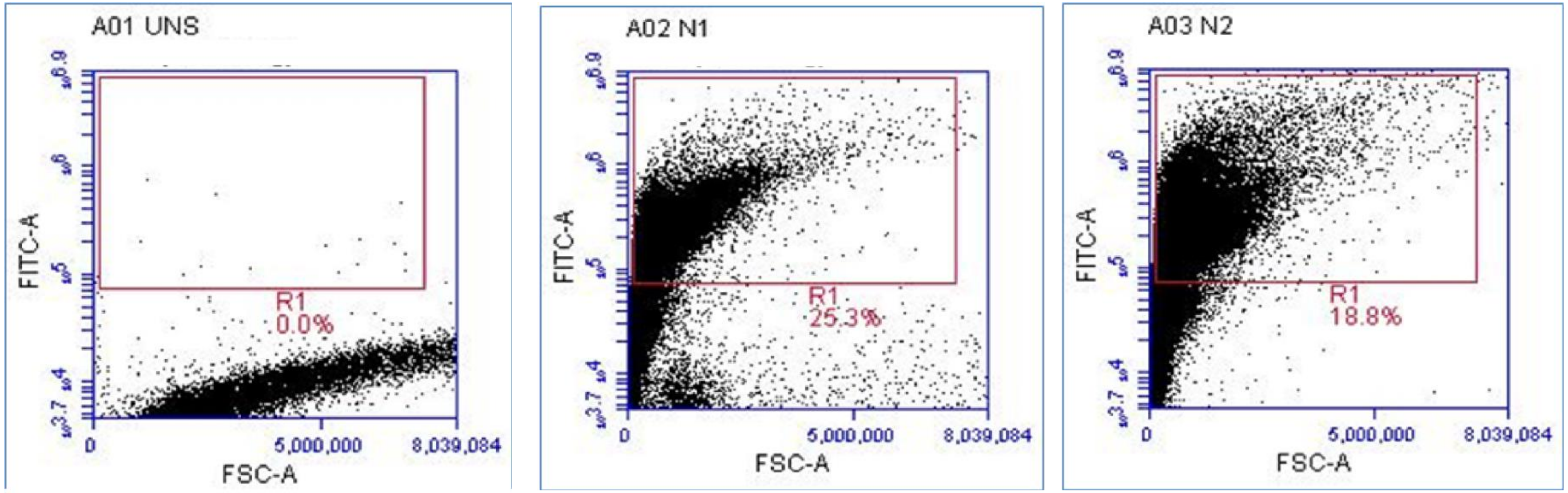

Merged all data for presentation

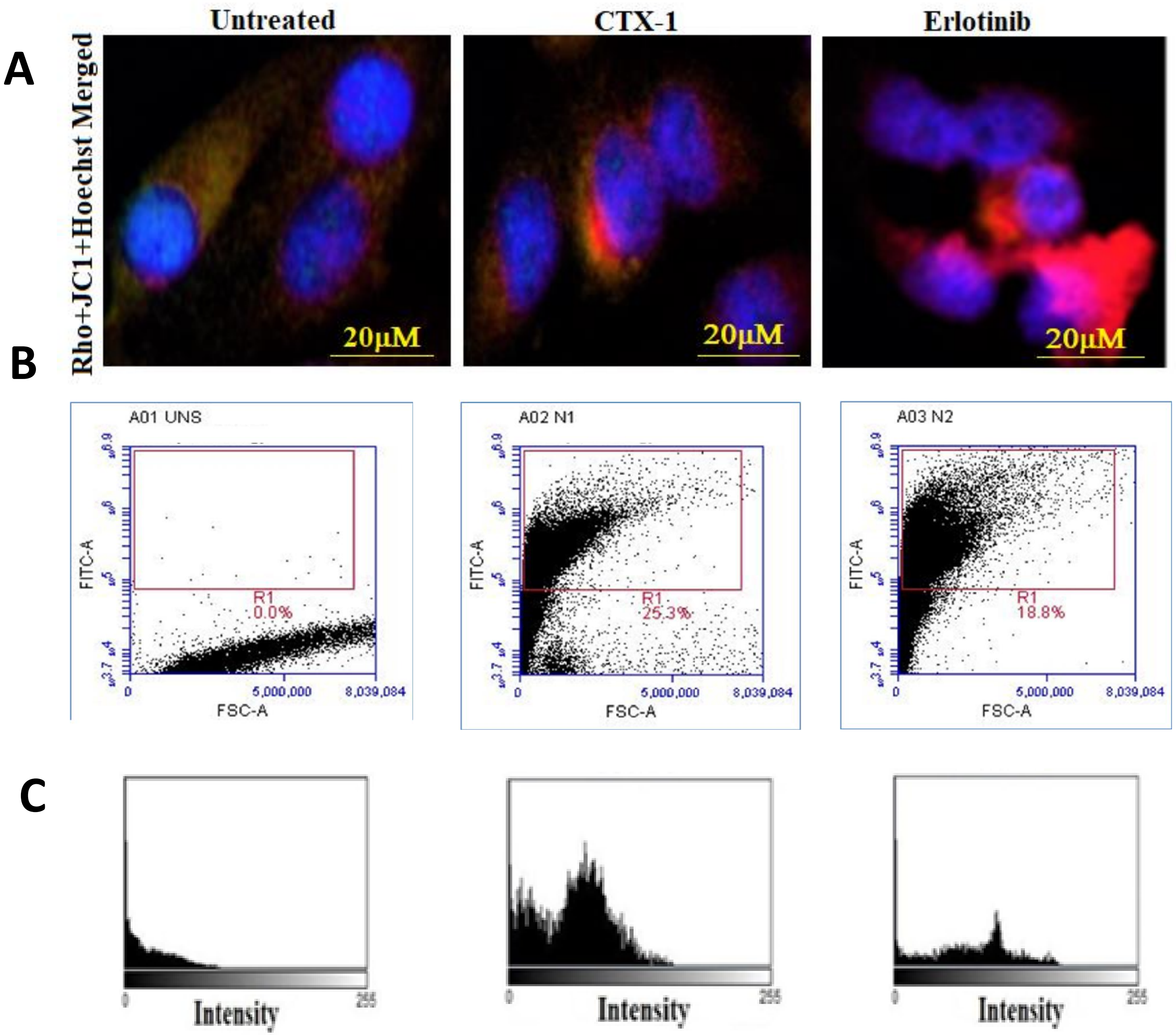

Supplement: Supplementary file 2 — Data S1. [file JCMM-28-e18263-s001.zip › R9-Mitochorndrial Transmembrane Potential.pdf]
