# Supplementary figures and images for: Decoding dynamic interactions between EGFR‐TKD and DAC through computational and experimental approaches: A novel breakthrough in lung melanoma treatment
Source: J Cell Mol Med. 2024 Apr 29;28(9):e18263. doi: 10.1111/jcmm.18263 (PMC11058330; doi:10.1111/jcmm.18263)

Processed the same as the raw data for the Presentation

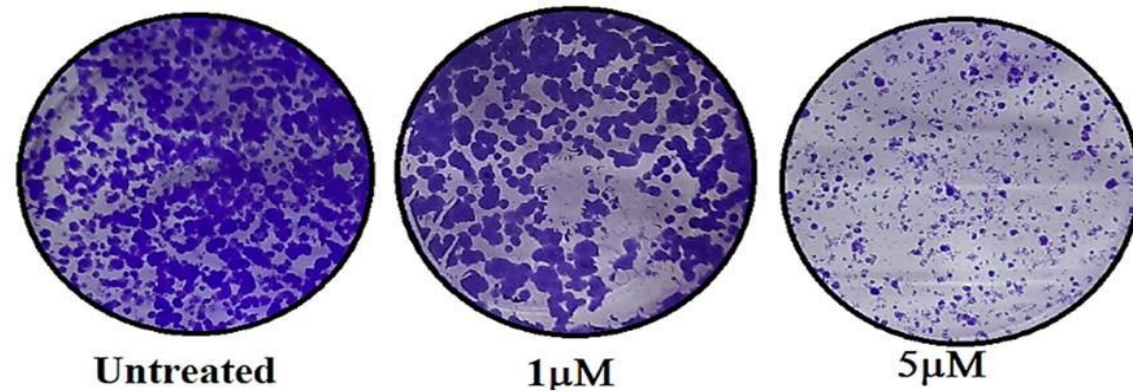

**A**

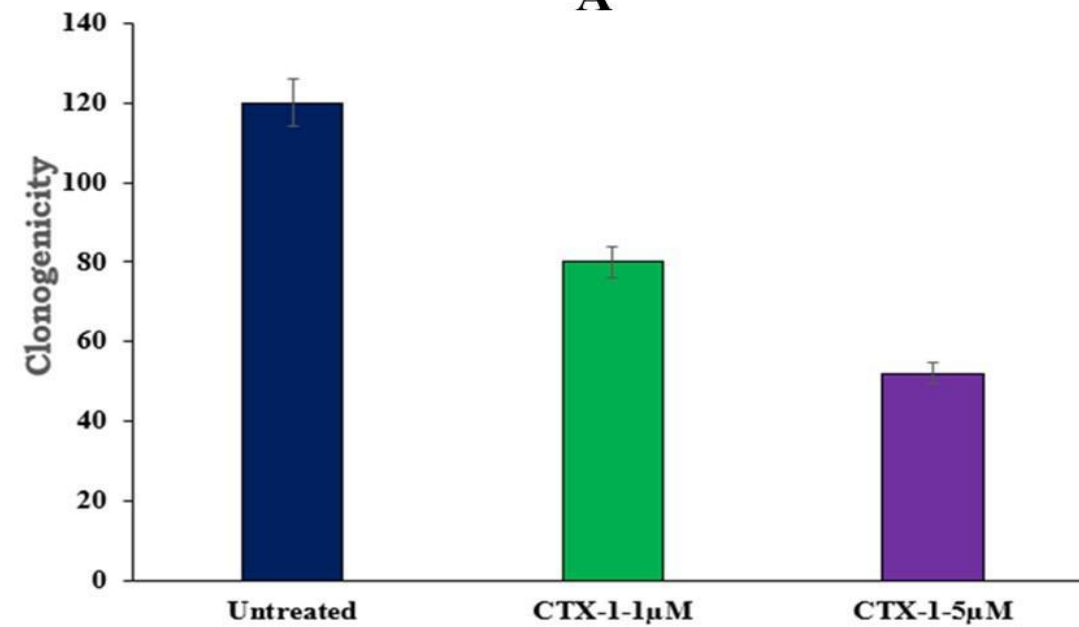

**B**

Supplement: Supplementary file 2 — Data S1. [file JCMM-28-e18263-s001.zip › R11-Colony Formation Assay.pdf]

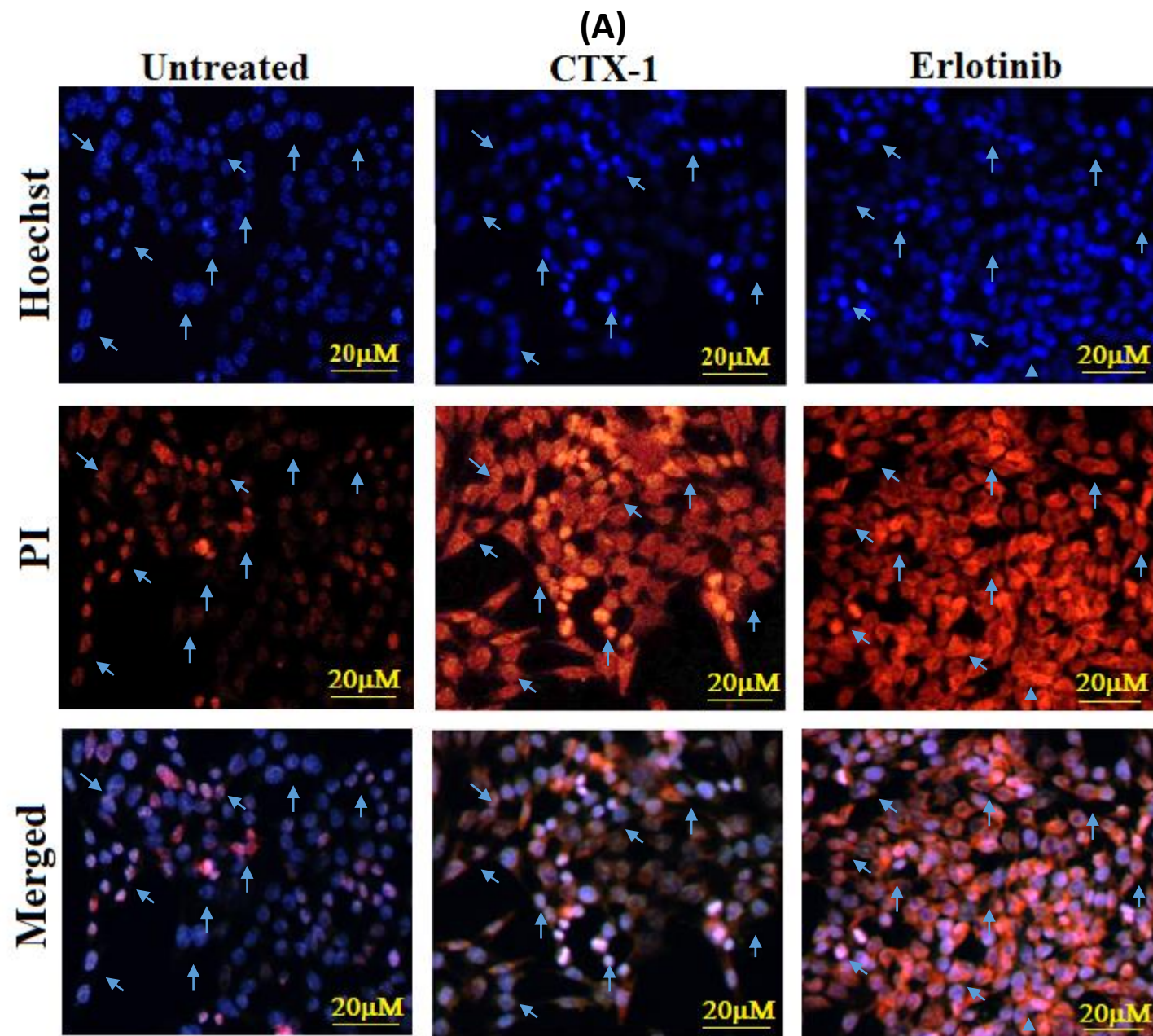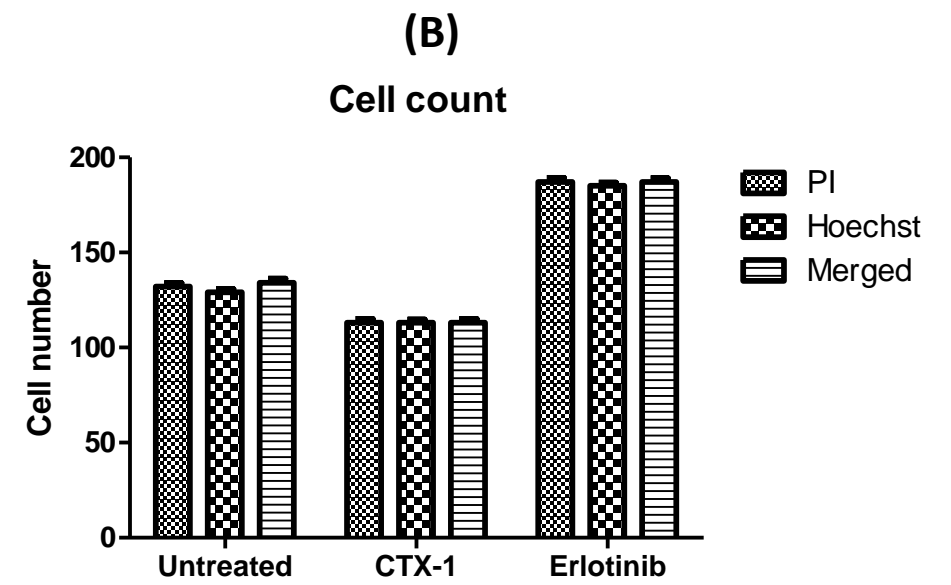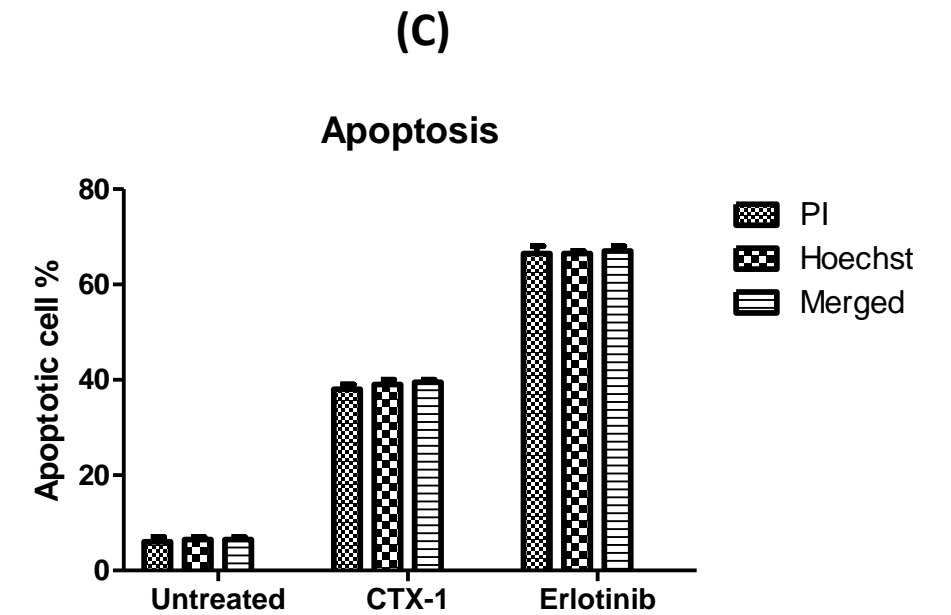

Supplement: Supplementary file 2 — Data S1. [file JCMM-28-e18263-s001.zip › R7-Apoptosis.pdf]

Raw data of cell cycle arrest

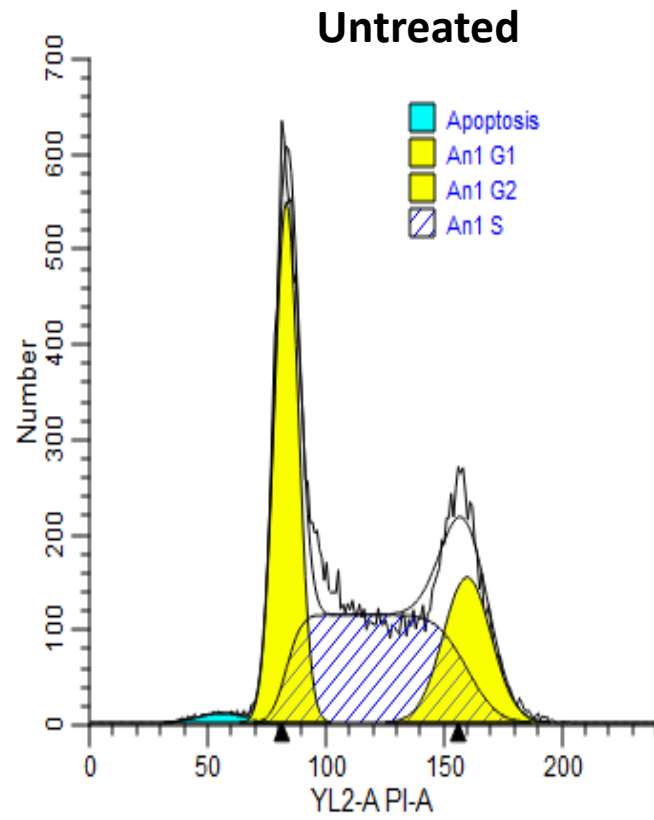

Processed same for presentation

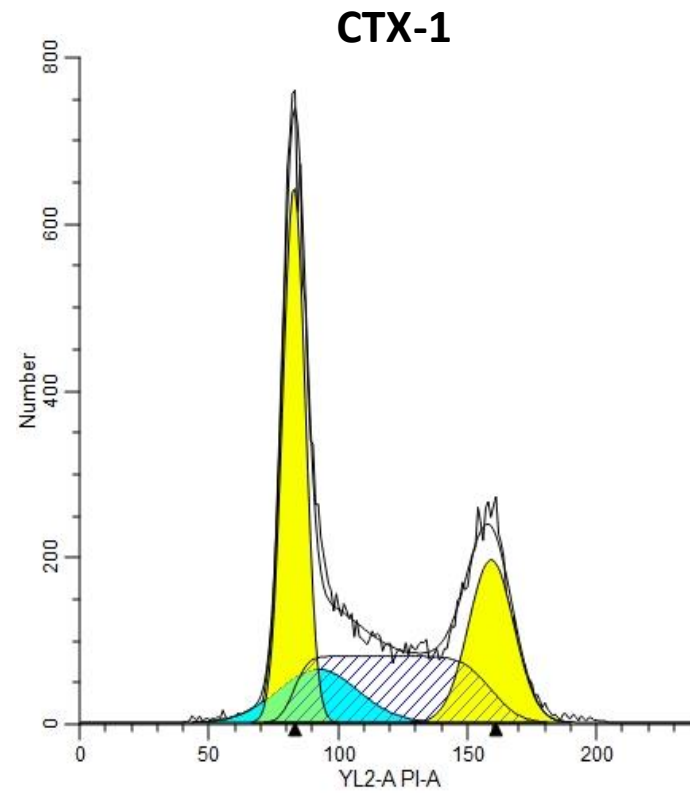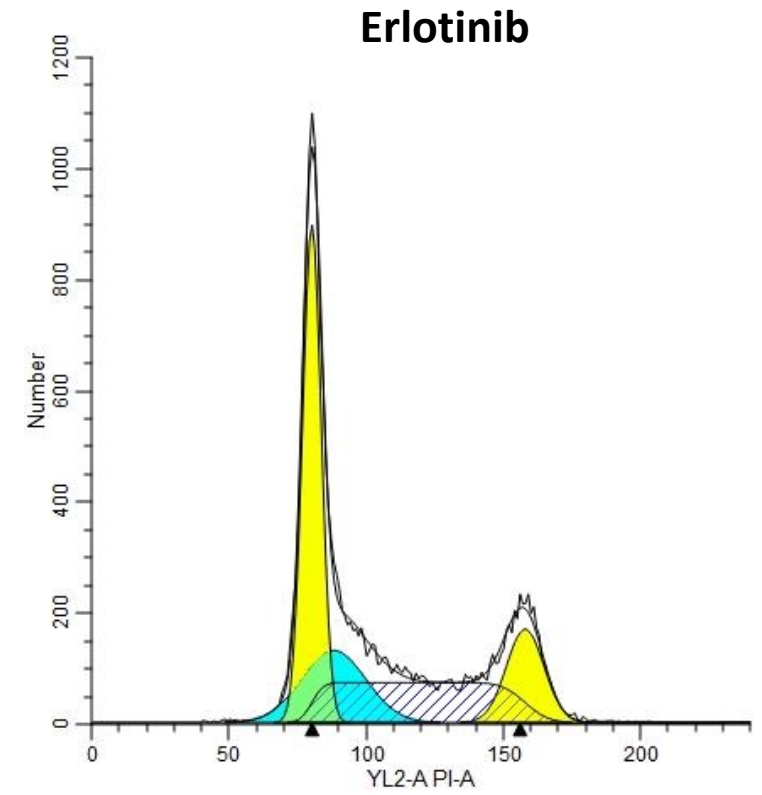

Supplement: Supplementary file 2 — Data S1. [file JCMM-28-e18263-s001.zip › R8-Cell_Cell_Arrest.pdf]
